# *De novo* transcriptome profiling of cold-stressed siliques during pod filling stages in Indian mustard (*Brassica juncea* L.)

**DOI:** 10.3389/fpls.2015.00932

**Published:** 2015-10-30

**Authors:** Somya Sinha, Vivek K. Raxwal, Bharat Joshi, Arun Jagannath, Surekha Katiyar-Agarwal, Shailendra Goel, Amar Kumar, Manu Agarwal

**Affiliations:** ^1^Department of Botany, University of DelhiNew Delhi, India; ^2^Department of Plant Molecular Biology, Central European Institute of TechnologyBrno, Czech Republic; ^3^Department of Plant Molecular Biology, University of DelhiNew Delhi, India

**Keywords:** *Brassica juncea*, transcriptome, cold stress, low temperature, silique, RNA-seq

## Abstract

Low temperature is a major abiotic stress that impedes plant growth and development. *Brassica juncea* is an economically important oil seed crop and is sensitive to freezing stress during pod filling subsequently leading to abortion of seeds. To understand the cold stress mediated global perturbations in gene expression, whole transcriptome of *B. juncea* siliques that were exposed to sub-optimal temperature was sequenced. Manually self-pollinated siliques at different stages of development were subjected to either short (6 h) or long (12 h) durations of chilling stress followed by construction of RNA-seq libraries and deep sequencing using Illumina's NGS platform. *De-novo* assembly of *B. juncea* transcriptome resulted in 133,641 transcripts, whose combined length was 117 Mb and N50 value was 1428 bp. We identified 13,342 differentially regulated transcripts by pair-wise comparison of 18 transcriptome libraries. Hierarchical clustering along with Spearman correlation analysis identified that the differentially expressed genes segregated in two major clusters representing early (5–15 DAP) and late stages (20–30 DAP) of silique development. Further analysis led to the discovery of sub-clusters having similar patterns of gene expression. Two of the sub-clusters (one each from the early and late stages) comprised of genes that were inducible by both the durations of cold stress. Comparison of transcripts from these clusters led to identification of 283 transcripts that were commonly induced by cold stress, and were referred to as “core cold-inducible” transcripts. Additionally, we found that 689 and 100 transcripts were specifically up-regulated by cold stress in early and late stages, respectively. We further explored the expression patterns of gene families encoding for transcription factors (TFs), transcription regulators (TRs) and kinases, and found that cold stress induced protein kinases only during early silique development. We validated the digital gene expression profiles of selected transcripts by qPCR and found a high degree of concordance between the two analyses. To our knowledge this is the first report of transcriptome sequencing of cold-stressed *B. juncea* siliques. The data generated in this study would be a valuable resource for not only understanding the cold stress signaling pathway but also for introducing cold hardiness in *B. juncea*.

## Introduction

The Brassicaceae family, which includes nearly 3500 species and 350 genera is one of the 10 most economically important plant families (Warwick and Black, 1991). Within the family, species of the genus *Brassica* comprise multiple vegetables (cabbage, broccoli, brussels sprout, cauliflower, turnip—*B. oleracea*), oilseeds (*B. rapa, B. juncea, B. napus*), and condiments (*B. nigra, B. carinata, B. juncea;* Branca and Cartea, 2011). *B. juncea* (*n* = 18) is an amphidiploid species derived from interspecific crosses between two diploid progenitor parents, *B. nigra* (*n* = 8) and *B. rapa* (*n* = 10) (Prakash and Hinata, 1980). It is grown as an oilseed crop in India (brown or Indian mustard), as a leaf vegetable in China, and as a condiment in western countries (Rakow, 2004). India is the third largest producer of rapeseed-mustard in the world after China and Canada. This crop accounts for nearly one-third of the edible oil produced in India, making it the country's key edible oilseed crop. The major impediments in harnessing the true yield potential of mustard are biotic stresses such as blight, aphids, white rust and abiotic stresses such as frost, high temperature, salinity, and drought.

Low temperature is one of the most intimidating abiotic stresses that affect plant growth and development, thereby limiting the distribution of crop species. Based on its intensity, cold stress can be broadly classified into chilling and freezing stresses. Exposure to temperatures below 0°C results in freezing stress, whereas chilling stress occurs at temperatures ranging from 0 to 20°C. Plants such as rice, maize and tomato that grow in tropical and subtropical regions are chilling sensitive whereas the plants from temperate region are chilling tolerant (Solanke and Sharma, 2008; Chinnusamy et al., 2007). Plants have the ability to acquire tolerance to chilling and freezing conditions if they are pre-exposed to non-freezing temperatures, through a process known as cold acclimation (Levitt, 1980). Cold acclimation helps plants to fine tune their metabolism and improve freezing tolerance by initiating signaling cascades that leads to several biochemical and physiological changes, including modification of membrane lipid composition and changes in gene expression (Shinozaki and Yamaguchi-Shinozaki, 1996; Thomashow, 1998; Gilmour et al., 2000; Chinnusamy et al., 2003). The altered gene expression leads to accumulation of several protective proteins such as antifreeze proteins (Griffith et al., 1997), late embryogenesis abundant (LEA) proteins (Antikainen and Griffith, 1997), heat shock proteins (HSP) (Wisniewski et al., 1996), cold-regulated (COR) proteins and various metabolites such as amino acids, soluble sugars, organic acids, pigments (Krause et al., 1999), polyamines (Bouchereau et al., 1999), and antioxidants (Hausman et al., 2000). These metabolites and proteins help in protecting plant membranes and prevent cell disruption during cold stress by stabilizing membrane lipids, proteins, maintaining hydrophobic interactions, ion homeostasis and scavenging the reactive oxygen species (ROS) (Hare et al., 1998; Gusta et al., 2004; Chen and Murata, 2008; Janská et al., 2010).

Spatial and temporal gene expression changes have traditionally been studied by comparing levels of steady state transcripts. With advancements of molecular techniques, it is now possible to generate information on alterations in transcripts levels at whole genome level. Using cDNA microarrays or whole genome arrays, the expression pattern of genes in response to chilling stress has been analyzed in *Arabidopsis*, rice, sunflower and several other plants (Seki et al., 2002; Rabbani et al., 2003; Fernandez et al., 2008). Seki et al. (2001) used a full-length cDNA microarray of 1300 *Arabidopsis* genes, and identified 19 *COR* (cold-regulated) genes, among which the newly identified genes were ferritin, a nodulin-like protein, LEA protein and glyoxalase. In another study, Fowler and Thomashow (2002) reported 306 *COR* genes using microarray for 8000 genes. Of these 306 genes, 218 were up-regulated and 88 were down-regulated and 45 of these *COR* genes were found to be expressing under the control of CBF1. Different ecotypes of *Arabidopsis* exposed to non-freezing cold stress exhibited different transcriptome level signatures (Barah et al., 2013). Transcriptome profiling of cold stress subjected 3-week-old *B. rapa* plants resulted in identification of genes encoding CBF/DREB like transcription factor, *ERD10, RD29A/COR78, COR47/RD17* (Lee et al., 2008).

Low temperature has a negative impact on different stages of plant reproductive development leading to premature abortion of seeds and fruits (Thakur et al., 2010). Likewise pod-filling stages in *B. juncea* are highly susceptible to frost stress injury thereby resulting in significant reduction in crop yield. Rajasthan, being the largest producer of mustard in India, contributes to 47.2% of the total production. Injury caused by episodic incidents of frost in semi-arid plains of Rajasthan has caused huge financial losses to the farmers. Cold waves and frost stress affects mustard cultivation in Rajasthan where night temperature in winters fall below −4.4°C. The loss in crop production due to frost stress in 2006 in Rajasthan alone was 344,400 tons resulting in total economic loss of Rs 5906 million (Prasada Rao et al., 2010). Since low temperature stress is a major limiting factor in *B. juncea* crops, it is significant to study the response of plants to stress and the underlying mechanism of tolerance. The first step in achieving this goal is to understand the plant response to non-freezing cold stress at the molecular level. To gain an in-depth knowledge of the global gene expression changes in developing siliques of *B. juncea* that were exposed to chilling stress, RNA-seq was employed to generate the transcriptome profile. Manually, self-pollinated siliques (5 DAP-30 DAP) of *B. juncea* were subjected to either short (6 h) or longer (12 h) durations of cold stress followed by RNA extraction, library construction and sequencing using Illumina's next generation sequencing platform. This is the first genome wide report of transcriptional response in *B. juncea* siliques that were exposed to cold stress. Deciphering the global gene expression changes in cold-stressed developing siliques, would be useful in understanding of the molecular pathway of cold stress and devising future strategies for enhancing low temperature tolerance in Indian mustard.

## Materials and methods

### Plant material and growth conditions

*B. juncea* var. Varuna seeds were obtained from National Seed Centre, IARI, New Delhi. Seeds were sown in the field at University of Delhi, South Campus during the growing season (October-March) of mustard. Plants were grown under field conditions and self-pollination was initiated at the time of flowering.

### Controlled self-pollination and stress treatment of *B. juncea* var. Varuna

*B. juncea* var. Varuna was manually self-pollinated with pollen derived from the same cultivar. All the open flowers and siliques were removed from the flowering twigs followed by emasculation of unopened buds. Fresh pollen was dusted on the exposed stigma followed by bagging to avoid cross-pollination. The manually self-pollinated siliques were subjected to cold stress at 5, 10, 15, 20, 25, and 30 days after pollination (DAP). Self-pollinated branches were excised and placed in a beaker filled with water and subjected to cold treatment at 4°C for 6 h and 12 h followed by immediate harvesting. Light conditions were maintained during cold stress to simulate natural conditions. For control samples, self-pollinated branches were excised and placed in water under field conditions. Self-pollinated siliques of different stages of development (5–30 days) were frozen in liquid nitrogen and stored at −80°C until further use. The temperature range in the field on the day of stress and harvesting was between 17°C (minimum) and −24°C (maximum). The entire experiment was designed such that control samples corresponding to different DAPs, were harvested on the same day. To study the anatomical stages of embryo development in *B. juncea* var. Varuna, resin sectioning was performed. The detailed procedure for staging and the results obtained is given in Supplementary Datasheet 1.

### Library preparation and data analysis

Total RNA was extracted from stressed as well as control siliques using Qiagen's plant RNA extraction kit as per the manufacturer's protocol. RNA-seq libraries were prepared utilizing TruSeq RNA sample preparation kit v2 (Illumina Inc., USA) as per the manufacturer's protocol and sequenced on HiSeq 2000 (Illumina, USA). The raw reads from sequencing run of all stages was subjected to a quality filtering utilizing “NGS QC toolkit” (Patel and Jain, 2012). Low quality reads whose 70% of the read length had phred score less than 20 (Q ≤ 20) were discarded followed by trimming of adapter sequences. Orphan reads remaining after applying these filtering criteria were also discarded. The QC filtered paired reads of all the samples were merged together into two files containing left and right reads, respectively. These merged files were used to reconstruct the transcripts using Trinity assembler with default parameters (Haas et al., 2013). The statistics of the assembled transcripts were measured utilizing TrinityStats.pl script of Trinity. QC filtered reads of each of the samples were aligned to the assembly using Bowtie2 aligner (Langmead and Salzberg, 2012) and quantification of each transcript was carried out with RSEM tool (Li and Dewey, 2011). Transcripts having less than 1 read count in all the samples were considered as misassembled or artifacts and hence discarded from the assembly. The statistics of the filtered assembly were again extracted using TrinityStats.pl script. Samples were normalized with the TMM normalization method (Robinson and Oshlack, 2010) of trinity and differentially expressed transcripts (with *p* < 0.001) were isolated using trinity differential expression module by applying a minimum cutoff of 2-fold change on log2 scale. Generation of heat map, Spearman correlation matrix, and cluster analysis of differentially expressed transcripts was performed using R-based tools. The reconstructed transcripts were annotated using FastAnnotator online server tool with default parameters (Chen et al., 2012a).

### Real time PCR validation

Total RNA was extracted from three independent biological replicates from stressed as well as control siliques (5–30 DAP) using Qiagen's plant RNA extraction kit as per the manufacturer's protocol. 10 μg of total RNA was subjected to DNaseI treatment with 1 U DNaseI (NEB, USA). The reaction was carried out at 37°C for 10 min followed by heat inactivation at 65°C for 10 min. 2.5 μg of DNase-treated RNA was used for cDNA synthesis with iScript reverse transcriptase (BioRad, USA) according to the manufacturer's protocol. The expression of actin gene (*ACT7*) was found to be stable in our transcriptome database and hence was used as the normalization control in real time PCR. The primers used for *ACT7* were forward primer: 5′ TGGGTTTGCTGGTGACGAT 3′ and reverse primer: 5′ TGCCTAGGACGACCAACAATACT 3′. Primers were designed for selected transcripts from transcriptome database and real time PCR was performed using SYBR green I master mix (Roche, GmBH) on CFX-Connect™ Real time system (BioRad, USA). Details of the primers are presented in Supplementary Table 1. Relative expression of the transcripts was calculated using ΔΔCt method (Livak and Schmittgen, 2001). Scatter plot and Pearson correlation coefficient was calculated in MS-excel using log2 values of qPCR and digital expression data.

The RNA-seq data discussed in this publication has been deposited in NCBI‘s Gene Expression Omnibus (Edgar et al., 2002) and are accessible through GEO Series accession number GSE73201 (http://www.ncbi.nlm.nih.gov/geo/query/acc.cgi?acc=GSE73201).

## Results

### *De novo* assembly of *B. juncea* silique transcriptome data

*B. juncea* var. Varuna plants were controlled self-pollinated at the time of flowering. Controlled self-pollinated siliques of *B. juncea* at different stages of development (5 DAP-30 DAP) were subjected to cold stress at 4°C for capturing the changes in gene expression. The siliques from control plants were also used to study the anatomical details of embryo development in *B. juncea* var. Varuna. Longitudinal sections of fertilized ovule showed first division of zygote at 5 DAP, first longitudinal division of embryo at 10 DAP, globular stage of embryo at 15 DAP, heart shape embryo at 20 DAP, torpedo stage of embryo at 25 DAP and cotyledonary immature embryo at 30 DAP (Supplementary Datasheet 1).

**Figure 1:**
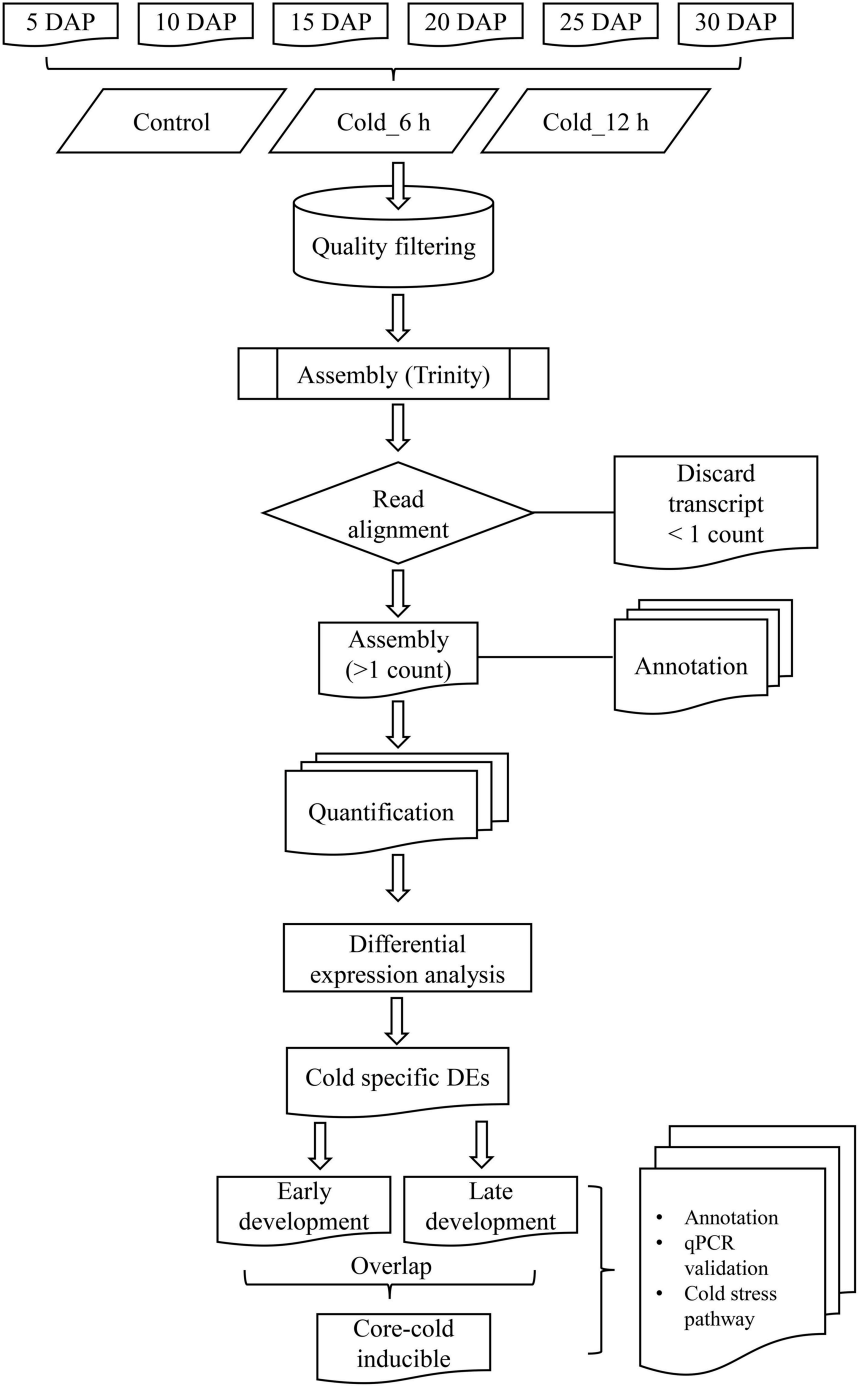
**Flowchart representing computational pipeline employed for analysis of transcriptome sequenced from ***B. juncea*** siliques**.

RNA-seq libraries were constructed from 18 different samples that encompassed six development stages (5, 10, 15, 20, 25, and 30 DAP) of *B. juncea* siliques, obtained by controlled self-pollination, and three different time points (0 h, also referred as control, 6 h and 12 h) of cold stress. Details of the experimental design and analysis pipeline are presented in Figure 1. As it was difficult to harvest enough embryos after controlled pollination, entire siliques were harvested for tissue collection. Sequencing of 18 libraries generated a composite of approximately 820 million reads, which were subjected to quality filtering using “NGS QC toolkit” (Patel and Jain, 2012). The adapter contaminated reads as well as reads whose 70% bases had Phred score less than 20 (Q ≤ 20) as well as orphan reads were discarded. This resulted in approximately 700 million high quality paired reads (Table 1).

**Table 1:** **Quality filtering and statistics of raw reads obtained in transcriptome libraries of ***B. juncea*** siliques exposed to low temperature for different durations**.

**S. No**.	**Sample**	**No. of raw reads**	**No. of HQ reads**	**Percentage (%)**	**Mapped reads**	**Percentage mapping (%)**
1	5 DAP_C	34,996,934	33,098,872	94.58	23,672,240	71.52
2	5 DAP_6 h	46,283,995	43,352,879	93.67	31,094,528	71.72
3	5 DAP_12 h	27,407,889	21,593,463	78.79	15,463,603	71.61
4	10 DAP_C	31,916,211	30,033,245	94.10	21,664,687	72.14
5	10 DAP_6 h	38,077,842	35,335,917	92.80	25,477,680	72.10
6	10 DAP_12 h	20,851,323	16,264,475	78.00	11,803,985	72.58
7	15 DAP_C	36,410,242	34,111,247	93.69	24,585,074	72.07
8	15 DAP_6 h	40,661,329	37,646,457	92.59	27,322,541	72.58
9	15 DAP_12 h	44,470,147	36,420,384	81.90	26,589,923	73.01
10	20 DAP_C	54,387,436	51,174,780	94.09	37,227,245	72.75
11	20 DAP_6 h	55,097,716	51,238,446	93.00	37,445,913	73.08
12	20 DAP_12 h	48,736,907	38,808,054	79.63	28,375,618	73.12
13	25 DAP_C	34,719,115	28,553,791	82.24	20,805,257	72.86
14	25 DAP_6 h	46,760,019	37,279,174	79.72	26,906,383	72.18
15	25 DAP_12 h	92,720,961	78,646,125	84.82	58,031,931	73.79
16	30 DAP_C	45,741,906	37,042,168	80.98	26,990,691	72.86
17	30 DAP_6 h	63,185,861	50,328,733	79.65	37,024,361	73.57
18	30 DAP_12 h	58,774,016	50,083,797	85.21	36,735,973	73.35

*Raw reads were filtered to remove adapter sequences and low quality reads using NGS QC tool kit. The percentage of reads retained after QC filtering is depicted in column 5*.

**Figure 2:**
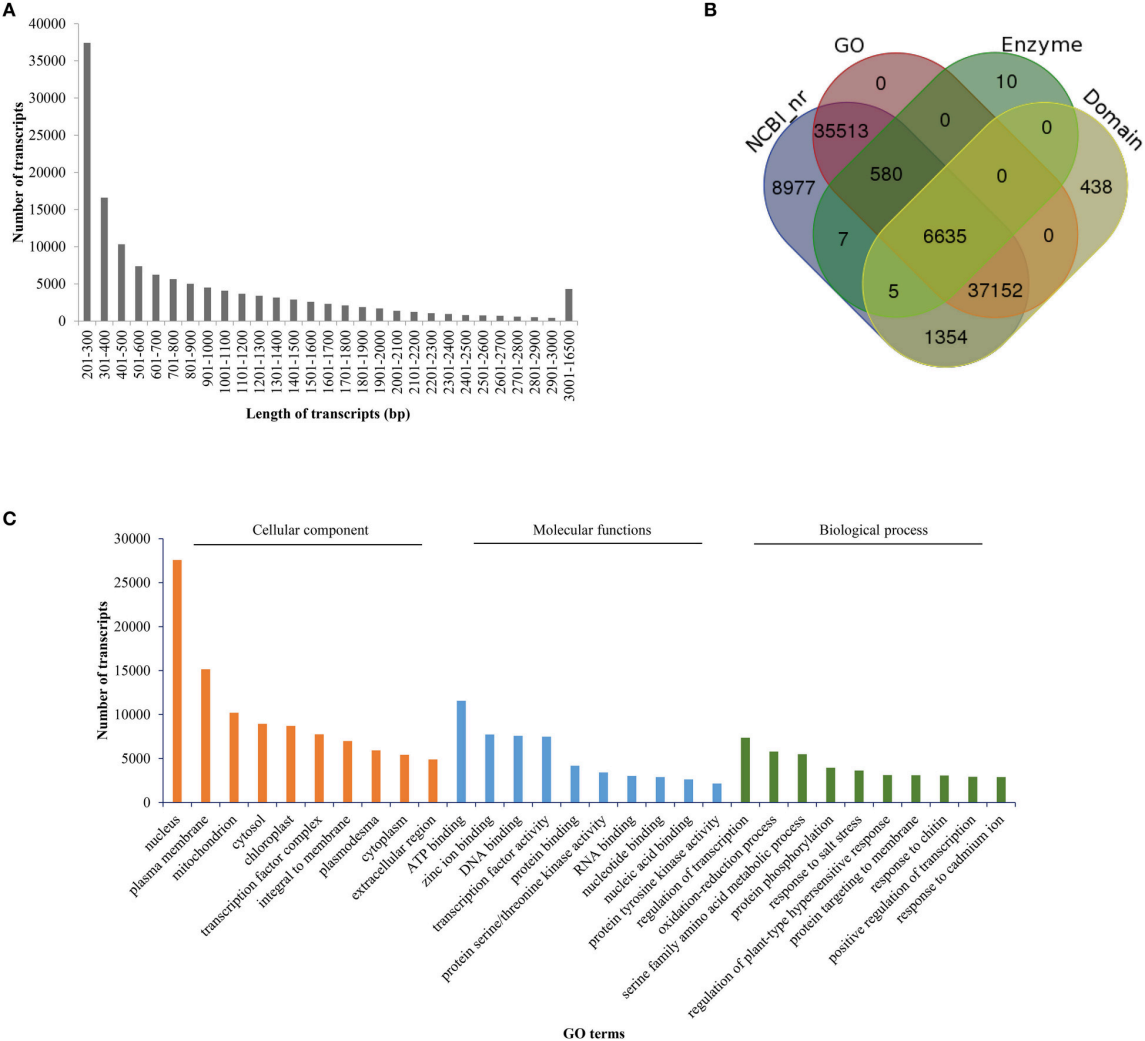
**Size distribution and gene ontology analysis of the assembled transcripts. (A)** Assembled transcripts were evaluated for their size distribution and the number of transcripts in each size range is presented. **(B)** Overlap in the distribution of annotated transcripts based on their databases hits. The LAST search was used to identify transcripts in NCBI nr database whose output was further annotated with Blast2GO for assigning gene ontology terms. PRIAM database and RPS blast were employed for identification of enzymes and conserved domains, respectively. **(C)** Gene ontology categorization of annotated *B. juncea* transcripts on the basis of cellular components, molecular functions and biological processes. The numbers of transcripts in the ten most enriched GO terms for each of the categories are shown.

The filtered HQ reads were subjected to assembly using the *de novo* assembler “Trinity” (Haas et al., 2013), which employs “de-Bruijn” graph approach (at a k-mer of 25 nucleotides) to assemble the reads into contigs. The preliminary assembly generated 212,124 contigs (hereafter referred to as “transcripts”). Combined length of these transcripts was 156 Mb and the N50 value of the assembly was 1202 bp. The obtained length of transcripts ranged between 201 and 14,199 bp and the GC content of assembled transcripts was found to be 41.92%. These transcripts also included isoforms and therefore represented 143,414 unigenes. The combined length of the unigenes was 83 Mb and had a N50 value of 822 bp. Average length of transcripts and unigenes (from the preliminary assembly) was found to be 737 and 577 bp, respectively. The preliminary assembly was subjected to assembly correction by aligning the HQ paired reads to the assembled transcripts with the help of Bowtie2 module of RSEM. More than 70% of the paired reads mapped to the transcripts of the preliminary assembly (Table 1). Quantification of each transcript was carried out using RSEM tool. Transcripts having expression value of less than 1 read count in all the 18 samples were removed from the assembled data. The final filtered assembly was reanalyzed for various parameters, including N50 value, transcript number, unigene number and average transcript length. Following assembly correction and filtering, 133,641 transcripts were retained, the combined length of which was 117 Mb. The average length of transcripts was 875 bp and the N50 value of the assembly was 1428 bp. While the maximum length of the transcript was found to be 16 kb, the minimum length was 200 bp. The distribution of transcript length is presented in Figure 2A. The 133,641 filtered transcripts represented 83,899 unigenes. The average length of the unigenes was 692 bp with a N50 value of 1225 bp (Table 2).

**Table 2:** **Assembly statistics of ***B. juncea*** transcriptome**.

**Parameters**	**Uncorrected**	**Corrected**
Number of transcripts	212,124	133,641
Number of unigenes	143,414	83,899
Assembly length (Mb)	156	117
Minimum transcript length (bp)	201	201
Average transcript length (bp)	737	875
N50 (bp)	1202	1428

### Annotation of assembled *B. juncea* siliques transcripts

To gain an insight on probable functions of each transcript, assembly was subjected to annotation analysis utilizing web-based tool “FastAnnotator”, which uses four different annotation protocols: (1) LAST (Local Alignment Search Tool) search to identify best hits in NCBI non-redundant (nr) database, (2) Blast2GO (https://www.blast2go.com/) to assign gene ontological (GO) terms, (3) PRIAM (http://priam.prabi.fr/) for identification of enzymes along with their EC numbers, and (4) RPS BLAST to predict functional domains in transcript sequences by searching the domain models (Pfamv26) from Conserved Domain Database (CDD; http://www.ncbi.nlm.nih.gov/cdd; Chen et al., 2012a). The LAST search against NCBI nr database resulted in annotation of approximately 67% transcripts (90,223 out of 133,640 transcripts) with a minimum *e*-value of 10^−5^ (Supplementary Datasheet 2). In addition to the NCBI-based annotation, 438 transcripts were annotated by domain search and 10 were annotated with enzyme database. Out of the 90,671 annotated transcripts, 79,880 transcripts could be associated with at least one GO term. Nearly, 7237 transcripts had at least one enzyme hit and 45,584 transcripts had >50% identity with previously reported protein domains (Figure 2B). Similarly, 8977, 438, and 10 transcripts exhibited exclusive hits to NCBI nr, domain and enzyme databases, respectively. The top 10 GO terms (associated with the annotated transcripts) in various biological processes, molecular functions and cellular components are presented in Figure 2C. The top GO terms in biological processes were regulation of transcription, oxidation-reduction, protein phosphorylation and stress responses. On the basis of localization in cellular components, maximum number of transcripts localized to nucleus followed by plasma membrane and mitochondria. ATP binding, zinc ion binding and DNA binding were the most enriched GO terms in the molecular function category.

### Identification of differentially expressed transcripts (DETs) in developing siliques

**Figure 3:**
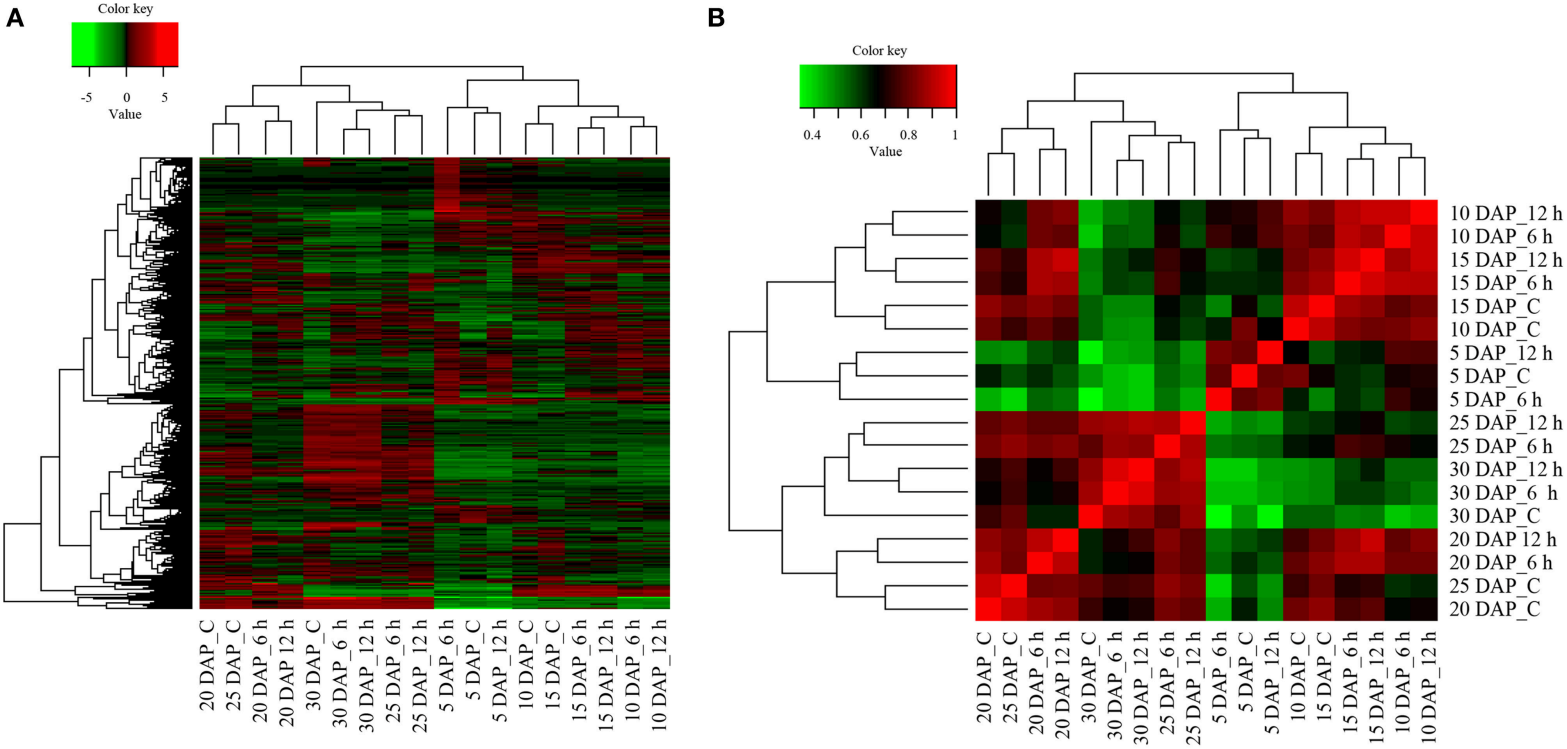
**Analysis of differentially expressed transcripts and their inter-relationships in ***B. juncea*** siliques**. **(A)** Heat map of differentially expressed transcripts in control and cold stressed *B. juncea* siliques at different stages of development. Differentially expressed transcripts identified by pair-wise comparisons of all the 18 samples were clustered together on the basis of their expression values and heat map was plotted with TMM-normalized FPKM values. **(B)** Spearman correlation matrix of all the samples indicating extent of similarity between each possible pair. All the 13,342 transcripts exhibiting differential expression were clustered together and Spearman correlation matrix was plotted on TMM-normalized FPKM values. The key for sample labels should be interpreted as x DAP_y, where x denotes number of days; DAP represents days after pollination and y denotes either unstressed siliques –“C” or duration of cold stress in hours –“h”.

Approximately, 13,342 transcripts exhibiting significant differential expression were identified by pair-wise comparisons (Supplementary Datasheet 3) in all possible combinations of the 18 samples. On the basis of expression profile, DETs were clustered to generate a heat map (Figure 3A) as well as Spearman correlation matrix (Figure 3B). Broadly, differentially expressed transcripts of 5, 10, and 15 DAP clustered in one clade whereas those of 20, 25, and 30 DAP clustered together in another clade, thereby enabling us to categorize and identify gene expression changes occurring either during the early (occurring in 5–15 DAP) or late (occurring in 20–30 DAP) stages of silique development. Transcripts of controls corresponding to 10 and 15 DAP clustered together. Similarly, transcripts present in controls of 20 and 25 DAP clustered together. Nonetheless, in most of the cases 6 h and 12 h cold stressed differentially expressed transcripts clustered together. The only exception was at 5 DAP whose control samples clustered with the 12 h cold stress samples, whilst 6 h samples was placed in a different sub-clade (Figures 3A,B). Following clustering, transcripts displaying similar expression pattern in response to cold stress were cataloged for each developmental stages separately and their expression pattern is depicted in Supplementary Figures 1–6.

**Figure 4:**
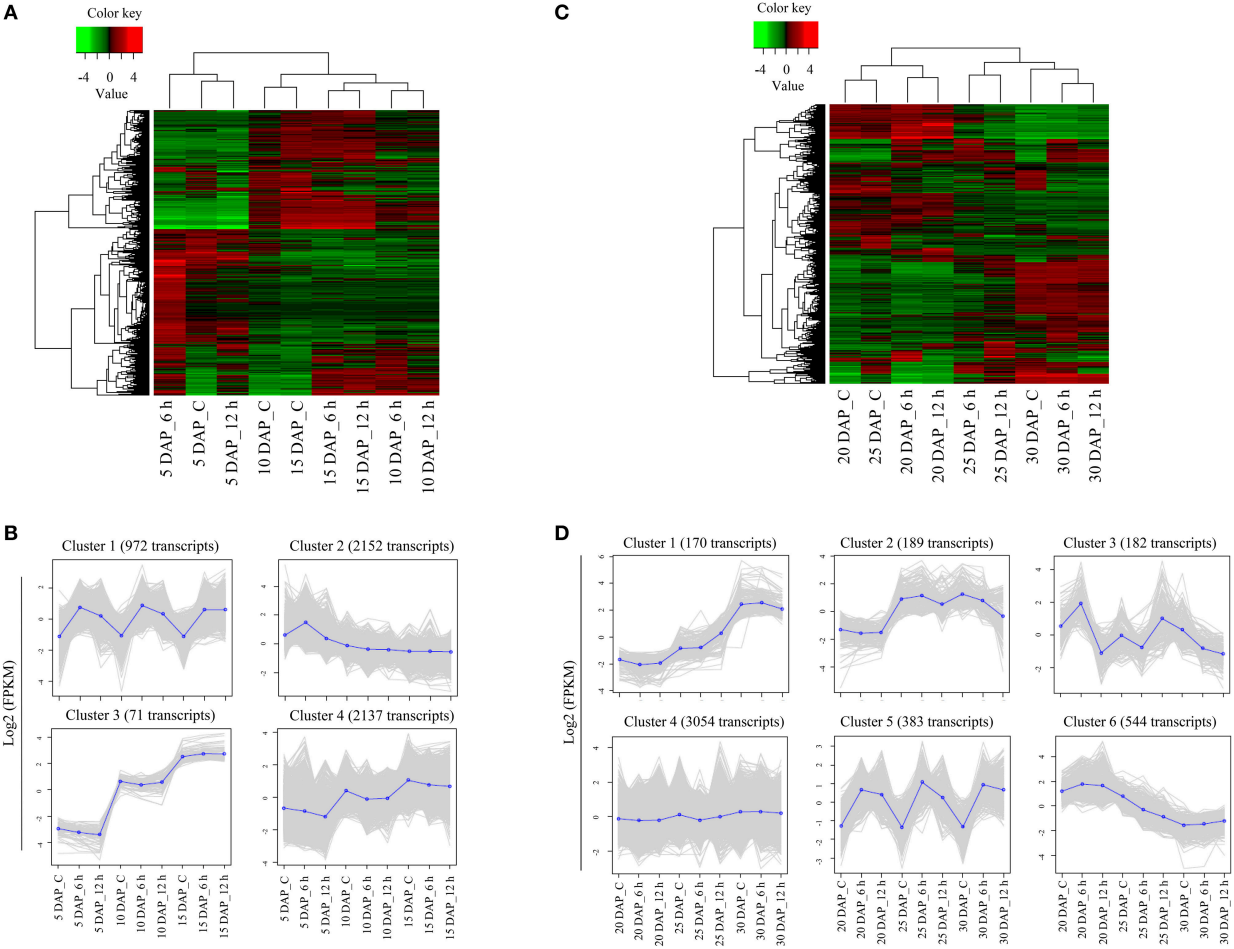
**Detailed analysis of differentially expressed genes in early and late stages of siliques exposed to cold stress**. Heat maps of differentially expressed transcripts in control and cold stressed *B. juncea* siliques at early **(A)** and late **(C)** stages of development. Differentially expressed transcripts identified by pair-wise comparisons of 9 samples of each stage were clustered together on the basis of their expression values and heat map was plotted with TMM-normalized FPKM values. Clusters of transcripts showing similar expression profile in early **(B)** and late **(D)** stages of silique development under control and stress conditions. The average expression of each cluster was plotted as dark blue line whereas gray lines represent expression of individual transcripts. The key for sample labels should be interpreted as x DAP_y, where x denotes number of days; DAP represents days after pollination and y denotes either unstressed siliques –“C” or duration of cold stress in hours –“h”.

The samples (control and cold stress treated) from 5, 10, and 15 DAP were considered as early stages of silique development. Detailed analysis of early stage samples identified 5332 transcripts exhibiting ≥2-fold difference in expression levels (on a log2 scale), which were subsequently used for generation of a heat map. As evident in Figure 4A, the hierarchical clustering of samples resulted in two parent clades where 5 DAP samples clustered under one clade whereas 10 and 15 DAP samples were part of another clade. Heat map and the associated clusters of expression profile (Figures 4A,B) showed that expression of a large number of transcripts is modulated significantly in response to cold stress. Based on the expression patterns of transcripts, four major categories comprising of 972, 2152, 71, and 2137 transcripts in clusters 1, 2, 3, and 4, respectively were identified (Figure 4B). Clusters 1 and 2 contained transcripts that were up-regulated by cold stress, whereas clusters 3 and 4 had transcripts that were developmentally regulated, as their expression remained unchanged under cold stress. Expression of the transcripts belonging to cluster 1 increased in response to both 6 h and 12 h of cold stress. More importantly, this trend was observed in all the initial stages (i.e., 5, 10, and 15 DAP) of silique development. Transcripts belonging to cluster 2 were also up-regulated by cold stress. However, the expression of these transcripts increased only at 6 h of cold stress in 5 DAP samples. Their expression was largely stable in all the other stages and time points.

The stages of silique development at 20 DAP, 25 DAP, and 30 DAP were categorized as late stages and these stages compositely had 4522 differentially expressed transcripts. Hierarchical clustering revealed that control samples of 20 DAP and 25 DAP clustered together. However, cold stress samples of 6 h and 12 h (at all the stages) were placed in the same cluster (Figure 4C). From the hierarchical clustering, we identified six major patterns of gene expression in the late stages of silique development (Figure 4D). Out of the six clusters, only two clusters (clusters 3 and 5) had transcripts that were induced by cold stress. The expression of transcripts in cluster 3 increased (w.r.t. to the corresponding controls) after 6 h of cold stress in 20 DAP and after 12 h of cold stress in 25 DAP. Noticeably, expression of transcripts in cluster 5 had a similar pattern as cluster 1 of early stage as these transcripts were induced by both 6 h and 12 h of cold stress in all the later stages of silique development.

### Identification and characterization of transcripts inducible by cold stress in *B. juncea* siliques

**Figure 5:**
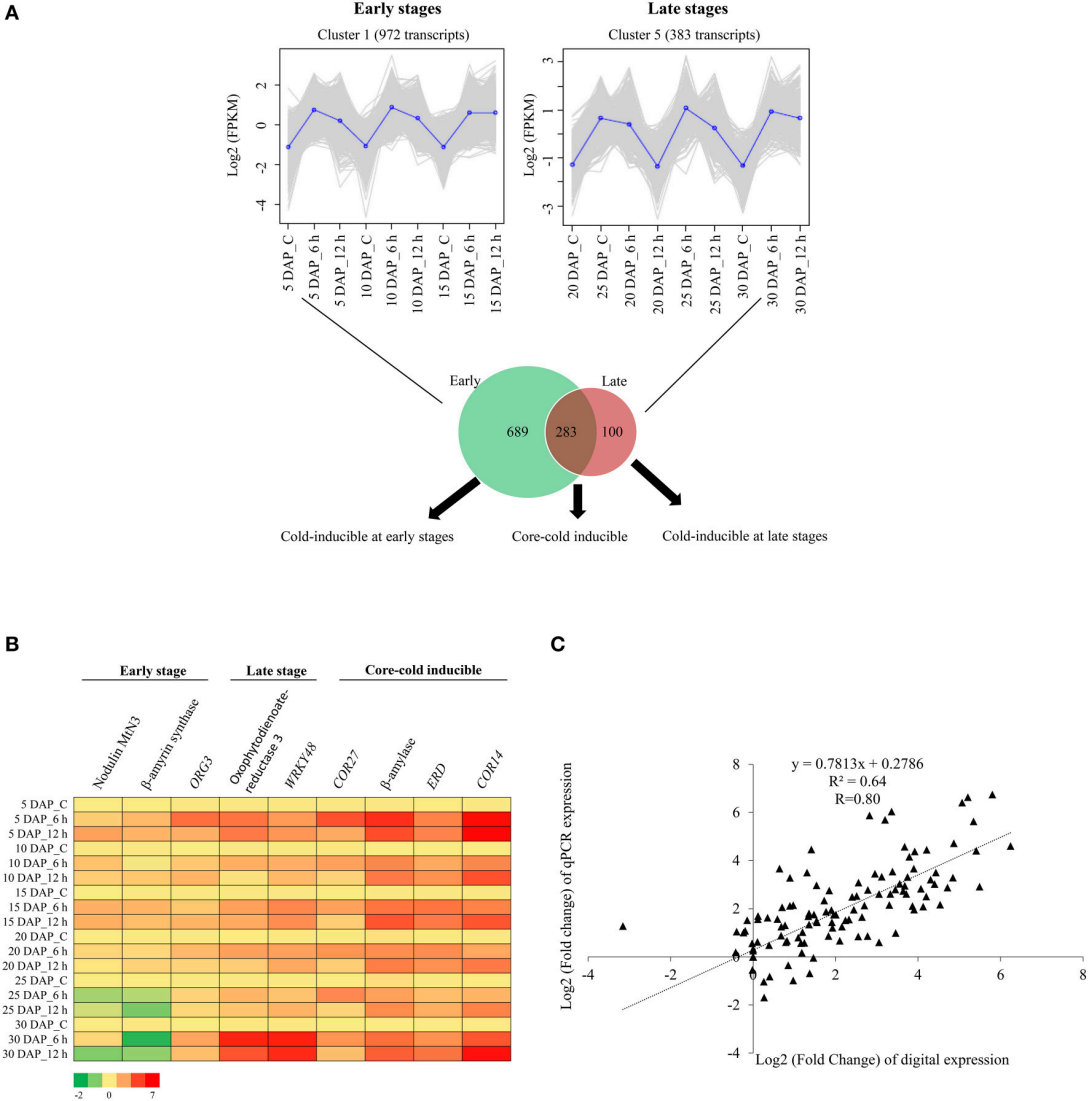
**Identification and validation of cold-inducible transcripts from early and late stages of silique development in ***B. juncea*****. **(A)** Transcripts of cluster 1 (early stage) and cluster 5 (late stage) were compared and candidates that were either specific or common to both the stages of silique development were identified. **(B)** Heat map showing relative abundance of selected cold-inducible transcripts belonging to the early, late and core subsets as determined by qPCR. **(C)** Scatter plot showing correlation between digital and qPCR expression profiles. Pearson correlation coefficient (R) was calculated between log2 fold change values of qPCR and digital expression. The key for sample labels should be interpreted as x DAP_y, where x denotes number of days; DAP represents days after pollination and y denotes either unstressed siliques –“C” or duration of cold stress in hours –“h”.

To identify the core group of genes that are induced in both the early and late stages of silique development, the transcripts from cluster 1 of the early stage (Figure 4B) was compared with those from cluster 5 of the late stage (Figure 4D). Comparison of 972 transcripts of cluster 1 with 383 transcripts of cluster 5 led to identification of 283 transcripts that were induced by cold stress in all the stages of embryo development. The 283 transcripts thereby constituted what is being referred to as the “core cold-inducible” transcripts. Additionally, this analysis revealed that 689 transcripts were specifically induced by cold stress in the early stages of silique development, whereas expression of 100 transcripts increased under cold stress, in the late stages of silique development. Results of this analysis are presented in Figure 5A and the list of the transcripts is provided in Supplementary Datasheets 4–6. To further assign relevance, transcripts from each of these subsets were annotated and their gene ontology terms in the biological processes category were identified (Supplementary Figure 7). Details of this analysis are presented below.

#### Annotation of subsets

Functional annotations of the three subsets were fetched from the annotated assembly. More than 70% of the transcripts from each of the subsets were annotated with different databases. Out of 689 transcripts in early inducible subset, 492 were annotated with different databases. Similarly, annotations were possible for 231 and 72 transcripts of core-cold inducible and cold-inducible subsets at late stages of silique development, respectively (Supplementary Figure 7). Majority of the transcripts in all the three categories were annotated by GO search as well as by the domain structure databases. However, none of the transcripts were annotated specifically from enzyme database alone.

Considering only the top 20 GO terms, gene ontology analysis revealed that some of the biological processes were exclusively associated with specific categories. For example GO terms, “MAPKK” and “protein targeting to membrane” associated exclusively with cold-inducible subset of early silique development. Similarly, the other subset, containing cold-inducible transcripts in the later stages of silique development, associated specifically with GO terms like “oxidation-reduction process,” “response to UV-B,” and “stomatal complex morphogenesis.” The GO terms “photoperiodic response,” “photo-morphogenesis,” “two-component signal transduction,” and “myoinositol hexaphosphate processes” were linked to the core-group transcripts. GO terms representing transcripts involved in response to various abiotic and biotic stresses like cold, wounding, fungal infection etc., were present in all the three subsets.

### Identification of TFs, TRs, and kinases inducible by cold stress in *B. juncea* siliques

Transcription factors (TFs) are proteins, which directly bind to the DNA sequence and modulate transcription of a gene, whereas transcriptional regulators (TRs) are proteins, which interact with other proteins to regulate gene transcription. Apart from these proteins, kinases are known for their role in relaying stress signals to the downstream signaling components. Therefore, an in-depth analysis of the transcriptome data was performed to identify TFs, TRs, and kinases that are inducible by cold stress in the three subsets of cold-inducible transcripts.

#### Transcription factors (TFs)

We identified 120 transcripts belonging to 22 TF families from the three subsets. These included members of various stress-responsive TFs such as WRKY, HSFs, MYBs, AP2-EREBs, NACs etc. Nineteen of the 22 families were detected in the early cold-inducible category and interestingly 12 of these were induced by cold exclusively during the early stages of silique development. Members belonging to 5 families of TFs were present in the later stages of development, however only 1 of these was exclusive to later stages. Maximum members (25) were detected for the Myb family; out of which 15 were cold-inducible exclusively in the early stages subset while another 10 were part of the core-group of transcripts. None of the Myb TF family member was inducible by low temperature in the later stages of silique development. The next major category of TF was AP2-EREBP type, which had 10 cold-inducible transcripts in the early group, 1 in late group and 4 in the core group (Table 3).

**Table 3:** **Major TF, TR and kinases family induced by low temperature belonging to three subsets (cold-inducible at early stage, cold-inducible at late stage, and core-cold inducible) of siliques development**.

**Type**	**Early stage specific**	**Late stage specific**	**Core-cold specific**
**TRANSCRIPTION FACTORS**			
ABI3VP1	1	ND	ND
AP2-EREBP	10	1	4
bHLH	8	ND	ND
bZIP	2	ND	ND
C2C2-CO-like	ND	ND	12
C2C2-Dof	5	1	ND
C2C2-GATA	1	ND	ND
C2H2	5	ND	ND
C3H	5	2	5
FHA	1	ND	ND
HB	4	ND	1
HSF	ND	1	ND
MADS	1	ND	ND
MYB	15	ND	10
NAC	7	ND	ND
PLATZ	2	ND	1
Sigma70-like	ND	ND	2
SBP	1	ND	ND
TCP	1	ND	ND
Tify	2	ND	ND
Trihelix	2	ND	ND
WRKY	6	1	ND
**TRANSCRIPTIONAL REGULATORS**			
ARID	1	ND	ND
GNAT	1	ND	ND
Orphans	7	ND	31
Pseudo ARR-B	ND	ND	6
TRAF	ND	ND	1
**KINASES**			
Calcium dependent protein kinase	1		
Domain of unknown function 26 (DUF26) kinase	1		
Legume lectin domain kinase	1		
Leucine rich repeat kinase I	1		
Leucine rich repeat kinase XI & XII	1		
Other protein kinase	3		
SNF1-related protein kinase (SnRK)	7		
Unknown function kinase	1		

*ND, not detected*.

The core-cold inducible subset consisted of well-known positive regulators of cold stress pathway like *CBF*s, AP2-EREB, constans-like 1, *DREB2-2*, and members of Myb TF family whereas late stage cold-inducible subset included *HSFA6b* and AP2-EREB members. Transcription factors were maximally represented in early stages of silique development. Members of WRKY family have been implicated in abiotic as well as biotic stress (Kayum et al., 2015). *WRKY23* and *WRKY46* were expressed under cold stress in early stages of silique development. SPL family of TFs is a functionally diverse set of proteins and is known to be involved in regulating plant growth and development (Preston and Hileman, 2013). In early stages of development, *SPL13* was found to be cold-inducible. Similarly, zeaxanthin epoxidase the first enzyme in ABA synthesis pathway (Schwartz et al., 1997) was inducible by cold stress in the early stages of siliques development. ABA is a plant hormone well known for its involvement in seed development as well as abiotic stress response (Nakashima and Yamaguchi-Shinozaki, 2013). There is a direct evidence of light regulated expression of *CBF*s in cold stress. Expression of *CBF*s is regulated by circadian-rhythm and depends upon red to far-red ratio of light (Fowler et al., 2005). In our data sets, it was found that phytochrome interacting transcription factor was inducible by cold stress during early developmental stages whereas circadian-rhythm regulating factor was up-regulated in core-cold inducible datasets.

#### Transcriptional regulators (TRs)

Transcriptional regulators are group of proteins, which interact with other proteins and affect transcription of genes. We were able to identify five families of cold-inducible TRs in developing siliques. Two families each were detected exclusively in early and core group of transcripts. None of the cold-inducible TR members were detected exclusively during late stages of silique development (Table 3). Members belonging to Orphan TRs, were abundantly represented in the core group of cold-inducible transcripts.

#### Kinases

Kinases play an important role in signal transduction by relaying the signal through protein phosphorylation. In our dataset, cold-inducible kinases were found only during the early phases of silique development. None of the kinases were up-regulated by cold stress either in later stages of development or were part of the core-cold inducible subsets. Families of kinases that are up-regulated by cold stress in the early development are presented in Table 3. A total of 16 transcripts encoding for kinases were identified out of which 7 belonged to the SNF1-related protein kinases.

### Expression profiling of differentially regulated transcripts by quantitative PCR (qPCR)

Nine transcripts representing all the three subsets (cold-inducible at early stage, core-cold inducible, and cold-inducible at late stage) were selected for validation of digital expression profile. Total RNA extracted from *B. juncea* siliques (5 DAP-30 DAP) that were subjected to 6 h and 12 h of cold stress, was used for quantification of the relative expression by qPCR. The heat map of relative expression for the 9 transcripts is depicted in Figure 5B and the corresponding bar graphs are given in Supplementary Figures 8–10. The expression pattern obtained by qPCR was in concordance with the expression pattern inferred by the RNA-seq data as shown by high Pearson correlation coefficient of 0.8 (Figure 5C). Three of the transcripts exclusive to early stages include β-amyrin synthase, ORG3-like transcription factor, and Nodulin MtN3 (*SWEET13*). The expression of Nodulin MtN3 (*SWEET13*) was up-regulated by cold stress in both 5 and 15 DAP of silique development, whereas β-amyrin synthase was inducible in 5, 10, and 15 DAP. ORG3-like transcription factor was inducible in cold stress subjected 5 DAP. These transcripts were not inducible or had a reduced expression in response to cold stress during the late stages (20, 25, and 30 DAP) of silique development. The cold-induced expression of Oxophytodienoate reductase 3 and WRKY transcription factor 48 (genes whose digital expression were high only in the late stages) was found in early stages also, however, their induction levels at 30 DAP were much higher than early stages. Four core cold-inducible transcripts (*COR27, COR14*, putative β-amylase, and early response to dehydration) were found to exhibit high expression at all the stages of silique development in response to cold stress.

**Figure 6:**
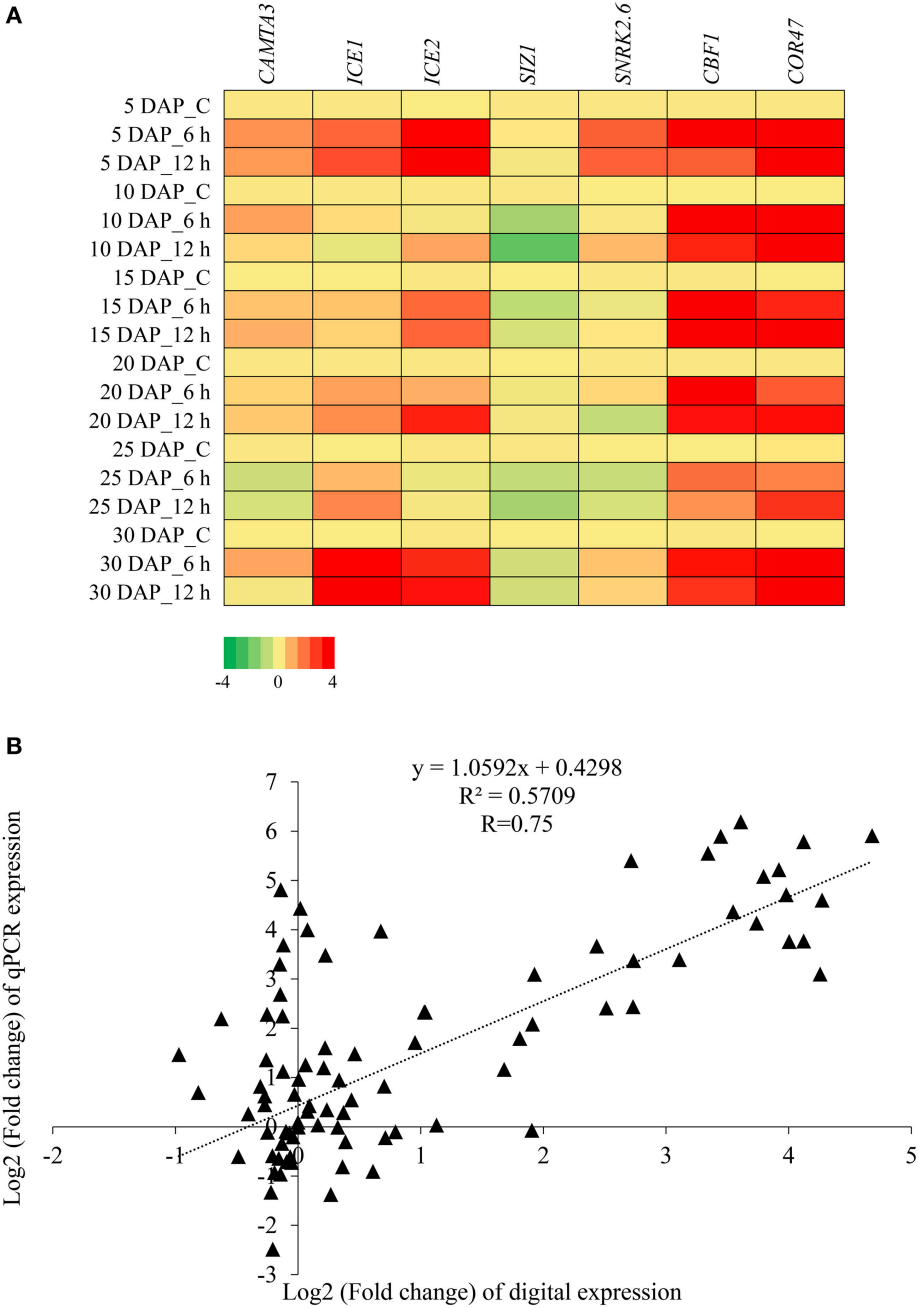
**Expression profiling of ***B. juncea*** transcripts homologous to cold-stress signaling pathway components. (A)** Heat map showing relative abundance of genes belonging to cold signaling pathway as determined by qPCR. **(B)** Scatter plot showing correlation between digital and qPCR expression profile. Pearson correlation coefficient (R) was calculated between log2 fold change values of qPCR and digital expression.

An additional set of seven transcripts, comprising known cold signaling pathway genes, were selected for expression analysis by qPCR. The heat map of relative expression profiles of the transcripts are depicted in Figure 6A and the bar graph of these genes are given in Supplementary Figure 11. *CBF1* and *COR47* were found to be inducible at all stages of silique development under cold stress, whereas *SnRK2.6* and *CAMTA3* were inducible at specific stages of silique development. The expression profiles of *ICE1* and *ICE2* were largely similar, and were induced after cold stress at 5 DAP and 30 DAP. However, increase in transcript level of *ICE2* was also observed in 15 and 25 DAP after cold stress. We failed to observe any significant increase in the expression of *SIZ1* in any of the developing stage on exposure to 6 h and 12 h of cold stress. The expression pattern obtained by qPCR was in agreement with the expression pattern inferred by the RNA-seq data. Pearson correlation coefficient (R) was 0.75 (Figure 6B).

## Discussion

The sowing period of mustard in India ranges from mid-October to first week of November (Shekhawat et al., 2012) and pod filling occurs at approximately 75–80 days after sowing (http://www.nuziveeduseeds.com/mustard-how-to-grow/). The minimum mean temperature during the pod filling stages ranges from 10 to 14°C (http://www.imd.gov.in/doc/Winter2010.pdf). As the early pod filling stages are sensitive to frost injury, it is important to understand the molecular response of developing siliques to low temperatures so that the gathered knowledge could be used for generating frost resilient *B. juncea* plants. To fully comprehend cold stress-mediated transcriptional response chilling stress was imposed to manually self-pollinated *B. juncea* siliques followed by RNA-seq. In several previous studies only vegetative parts of plants were exposed to cold stress and although it is likely that cold stress during the vegetative phase of plant growth affects the reproductive phases, we imposed cold stress to the developing siliques of *B. juncea* as cold and frost stress largely affects post-fertilization stages. Our study allowed us to focus on identification of specific changes in gene expression in post-fertilization stages, which define the yield under stress conditions. Nonetheless, it would be worthwhile to identify genes that impact developmental programming in plants exposed to cold stress during the vegetative phase. In the present study excised self-pollinated branches of field-grown mustard plants were subjected to low temperature in a cold chamber. To normalize the wounding response, self-pollinated branches were excised and placed in a beaker containing water under field conditions and this tissue was used as control. Cold stress was imposed for either 6 h or 12 h to identify gene expression changes in transcription factors and kinases, which are normally induced early as well as their downstream target genes that are up-regulated after a longer period of stress. With the help of transcriptome sequencing we were able to identify subsets of cold responsive genes that are expressed in early and/or late stages of silique development. As reference genome for mapping the sequencing reads was not available, paired-end sequencing was performed to obtain an improved de *novo* assembly. In addition to the sequencing length, it is important that libraries should be sequenced with adequate depth so as to capture low abundance transcripts. As per the estimates published in a recent report, sequencing of >200 million paired-end reads can optimally discover rare transcripts and their isoforms in human genome (Sims et al., 2014). In the current study, approximately 700 million purity-filtered reads were generated, collectively from all the libraries in *B. juncea* (whose genome size is estimated to be one third of the human genome), and we believe that this depth is sufficient for discovering even the rare isoforms. Assembly, of transcripts was performed using trinity assembler, which resulted in 133,641 transcripts and composed 117 Mb of sequences.

Approximately 13,000 differentially regulated transcripts were identified by pair-wise comparison of all the 18 samples. Based on the expression profile of DETs, two distinct clades were observed- one comprising of 5, 10, and 15 DAP samples and another of 20, 25, and 30 DAP samples. Because of the clustering pattern, we categorized gene expression changes according to the early and late phases of silique development. It was also observed that cold stress subjected samples (i.e., 6 h and 12 h) clustered together and were distinct from control samples. We identified multiple transcripts that were regulated by cold stress. Notably genes that code for proteins like dehydrin, *DREB1B*, early response to dehydration, low-temperature induced (Lti), glycine rich RNA binding protein, calcineurin B-like (CBLs), CBL-interacting protein kinases (CIPKs) etc. were up-regulated by low temperature stress. In several studies these proteins were shown to be involved in cellular response to cold stress (Gilmour et al., 1998; Fowler and Thomashow, 2002; Maruyama et al., 2004).

To identify the core components of cold stress signaling in developing siliques, we compared the transcripts that are cold-inducible of the early stages (5, 10, and 15 DAP) with those of late stages (20, 25, and 30 DAP). A total of 1072 transcripts were inducible by cold stress out of which 283 transcripts were detected from both early and late stages of silique development. This subset was therefore considered as the core group of cold-inducible transcripts. In addition, 689 transcripts and 100 transcripts were induced specifically in early and late stages of silique development respectively. These transcripts were therefore categorized as either early or late components of the cellular response to cold stress in developing siliques of *B. juncea*. To gain a better understanding about the role of these genes, we annotated them on the basis of their homology and more than 70% of the transcripts were annotated using existing databases. Multiple GO categories whose genes were involved in various abiotic and biotic stresses were highlighted in all the three subsets. Transcripts belonging to GO category “Circadian Rhythm” constituted the second largest number in the core group of transcripts. It is now well-known that circadian rhythmicity and cold stress mediated gene expression are interconnected (Espinoza et al., 2008; Dong et al., 2011; James et al., 2012; Maibam et al., 2013). Our results also show that transcripts belonging to circadian rhythmicity are cold-inducible throughout the silique development phases. Additionally, some of the transcripts of circadian rhythmicity were inducible by cold specifically in the later phases of silique development.

Another major GO category whose transcripts were inducible by low temperature was “long-day photoperiodism.” Regulation of cold-inducible genes and freezing tolerance by light quality and duration have been previously reported in *Arabidopsis* (Franklin and Whitelam, 2007; Lee and Thomashow, 2012) and therefore, cold-induction of transcripts involved in photoperiodism is an additional evidence that these processes are intimately linked. A GO category whose transcripts were specifically up-regulated in the later stages of cold stress was “oxidation-reduction” process. One of the harmful effects that cold stress imposes is generation of ROS. Some of the genes that counteract the effects of ROS are cold-inducible (Lee et al., 2002; Zhu et al., 2007; Shi et al., 2014). Though generation of ROS is a function of stress, the enrichment of cold-inducible transcripts involved in oxidation-reduction process only in the later phases (which are exposed to similar cold stress as the early stages) is quite intriguing and requires further investigations.

A total of 120 transcripts belonging to 22 different TF (transcription factor) families were identified from the 1072 cold-induced transcripts. A large number of the TF transcripts (79) were cold-inducible specifically during early stages of silique development. These stages are vulnerable to the low temperature stress and therefore cold induction of kinases in these stages is possibly a mechanism to produce protective proteins by phosphorylation-mediated activation of the upstream TFs. During later stages of embryo development, LEA proteins start accumulating (Battaglia et al., 2008), and provide protection to the cells from ensuing desiccation. The core-cold-inducible subset has 4 members of AP2/EREBP family, which included one *CBF (DREB1)*, two *DREB2* members, and a single ERF. The core group also has 4 and 8 members of C2C2-constans like and C2C2-dof like TF family members, respectively, which are known to play regulatory roles in *Arabidopsis* in response to cold (Mikkelsen and Thomashow, 2009). The major family of TF enriched in core-cold (10 transcripts) as well as early stage cold-inducible (15 transcripts) subset is MYB TF family. The role of MYB genes in regulating genes of cold stress signaling pathway and in governing tolerance to cold stress is well documented (Vannini et al., 2004; Agarwal et al., 2006; Dai et al., 2007). CIRCADIAN CLOCK-ASSOCIATED 1 (CCA1), also belongs to MYB TF family and positively regulates expression of *CBFs* in *Arabidopsis* (Dong et al., 2011). Various transcripts coding for other TFs like NAC and WRKY were also identified from early stage cold-inducible subset. Transcript having homology to *AtHSFA6b* was induced by cold stress in later stages. These results are similar to the unpublished work from our lab where we found that *Arabidopsis HSFA6b* was inducible by low temperature.

Interestingly, it was found that a significant number of transcripts coding for transcriptional regulators (TRs) were inducible by low temperature. Some of the transcripts displayed inducibility in all the stages, whereas others were inducible only in the early stages. None of the TRs were cold-inducible exclusively in the later stages. One of the cold-inducible TR in early stages belonged to the GNAT (Gcn5-related N-acetyltransferase) family. Though it has been shown that CBF1 interacts with GCN5 (Stockinger et al., 2001; Mao et al., 2006), a direct role of GCN5 in promoting acetylation at COR promoters was not observed (Pavangadkar et al., 2010). Nonetheless, identification of a highly cold-inducible homolog of GNAT in *B. juncea* indicates that histone acetylation plays a critical role in modulating gene expression during cold stress. Involvement of histone modifications in cold stress is further supported by identification of a cold-inducible transcript of ARID (AT-rich interaction domain) family, members of which function as chromatin remodelers and histone demethylases (Tu et al., 2008; Lu and Tobin, 2011; Lin et al., 2014). Apart from TRs and TFs, multiple families of kinases induced by cold stress specifically during the early stages of embryo development were identified. The largest group of cold-inducible transcripts belonged to SnRK family. Members of SnRK families are involved in plant response to abiotic stresses (Ma et al., 2009; Umezawa et al., 2009; Vlad et al., 2009). Though, SnRK3 family members are routinely linked with abiotic stress response (Kim et al., 2003; Fujii and Zhu, 2009; Fujii et al., 2011), a recent study showed that the SnRK2 family member OST1 kinase phosphorylates ICE1, thereby increasing its stability and freezing tolerance in *A. thaliana* (Ding et al., 2015).

Quantitative PCR was employed to validate the expression pattern congregated from RNA-seq data. Nine transcripts representing all the three stages (cold-inducible at early stage, core-cold inducible and cold-inducible at late stage) were selected for qPCR analysis. The 4 transcripts from core-cold inducible category included *COR27, COR14*, putative β-amylase, and early responsive to dehydration. Similar to the digital expression data, these genes were found to be inducible by cold stress in all the stages of silique development. Mikkelsen and Thomashow (2009) identified *COR27* along with *COL1* to be cold-inducible and as well as regulated by circadian clock. *COR14* is a cold stress regulated gene isolated in barley (Crosatti et al., 1995). Higher levels of *COR14* were observed in cold-tolerant variety during cold acclimation as compared to susceptible variety (Cattivelli et al., 1995). β-amylase hydrolyzes α-1,4 glycosidic linkages of polyglucan chains at the non-reducing end to produce maltose which in turn protects membranes, proteins, and photosynthetic electron transport chain during severe temperature stress (Kaplan and Guy, 2004). Cold stresses and dehydration result in the up-regulation of stress-induced genes such as RD (responsive to dehydration), ERD (early responsive to dehydration), COR (cold regulated), LTI (low-temperature induced), and KIN (cold-inducible). During cold acclimation, *DREB*s bind to the promoter region of the downstream target genes such as *RD29A, ERD10, COR15A, RD17*, and *Kin2*, subsequently resulting in low temperature tolerance (Seki et al., 2002). Enhanced expression of *COR, RD*, and *ERD* have been observed in transgenic *Arabidopsis* plants overexpressing *DREB1A* when exposed to low temperature (Liu et al., 1998; Kasuga et al., 1999). Transcripts selected from “cold-inducible at early stage” subset include Nodulin MtN3 family protein, β-amyrin synthase (bAS), and Transcription factor ORG3-like. Members of nodulin MtN3 family are polytopic membrane proteins having MtN3/saliva domain and some of the members of these family like *AtSWEET15* are cold-inducible, however their functional role in cold stress has not yet been characterized (Seo et al., 2011; Yuan and Wang, 2013). β-amyrin synthase is involved in biosynthesis of the secondary metabolite β-amyrin, which serves as an intermediate in the synthesis of triterpene glycosides associated with plant defense (Kemen et al., 2014). We found that bAS gene is mildly inducible by low temperature stress, which is in agreement with its previously reported stress-inducibility in *Bruguiera gymnorrhiza* and *Glycirrhiza glabra* (Basyuni et al., 2009; Nasrollahi et al., 2014).

Oxophytodienoate-reductase 3 and WRKY transcription factor 48 were shortlisted from “cold-inducible at late stage” subset. Oxophytodienoate-reductase 3 catalyzes reduction of double bonds in unsaturated aldehyde and ketone leading to production of jasmonic acid, which is involved in combating various abiotic and biotic stresses (Creelman and Mullet, 1995; Schaller et al., 2000). WRKY transcription factors are involved in plant responses to various biotic and abiotic stresses (Chen et al., 2012b). Specific members of *Arabidopsis WRKY* TF family are regulated during early stages of cold stress (Bakshi and Oelmuller, 2014). Several WRKY family genes were found to be responsive to cold stress in soyabean, *Vitis vinifera*, and barley (Marè et al., 2004; Zhou et al., 2008; Wang et al., 2014).

Attempts were made to study the expression of known cold signaling pathway genes. Primers, corresponding to sequences of *CBF1, CAMTA3, SIZ1, ICE1, ICE2, SnRK2.6*, and *COR47* were designed and expression of the above genes in cold stress subjected *B. juncea* siliques was quantified using real time PCR. *CBF*s bind to CRT/DRE cis-elements and induce the expression of downstream cold-regulated genes (Gilmour et al., 1998; Steponkus et al., 1998; Cook et al., 2004). Levels of *B. juncea CBF1* increased on exposure to both 6 h and 12 h of cold stress, however *CBF1* transcripts accumulated to lower levels at 12 h of cold treatment as compared to 6 h in all the development stages. This is in sync with previous reports where early induction of upstream transcription factors was observed after cold stress in *A. thaliana* (Chinnusamy et al., 2010). Targets of *CBF*s include member of Cold Regulated *G*enes (*COR*s). One of the cold-regulated genes, *COR47*, was validated and its transcript was up-regulated at all the stages of silique development after imposition of cold stress. Doherty et al. (2009) showed the dependence of *CBFs* expression on *CAMTA3* as cold-induced accumulation of *CBF*s was substantially reduced in *camta3* lines. *CAMTA3* was identified as a cold-induced gene in our transcriptome datasets, which was subsequently validated by qPCR. Moreover, a direct correlation in the levels of *CAMTA3* and *CBF1* support the previously proposed link between the two genes. At all the stages of silique development, expression of *CAMTA3* was induced by cold stress except at 25 DAP where lower levels with respect to control were observed. Similarly, cold-induced expression of *CBF1* was also considerably low at 25 DAP as compared to other stages. The decrease in *CAMTA3* levels at 25 DAP did not necessarily obliterate cold-inducibility of *CBF1*, thereby indicating that in addition to *CAMTA3* other TFs might also play a role in cold stress mediated regulation of *CBF1*. *ICE1* is the master regulator of cold stress which enhances the expression of *CBF*s and downstream *COR* genes, eventually conferring freezing tolerance (Chinnusamy et al., 2003). *ICE2*, a homolog of *ICE1*, also induces expression of *CBF1/DREB1B* and confers increased freezing tolerance in *Arabidopsis* (Fursova et al., 2009). We observed increased expression of *ICE1* and *ICE2* in *B. juncea* siliques exposed to low temperature stress. *SnRK2.6* also known as open stomata 1 (OST1) is a Ser/Thr protein kinase involved in ABA signaling. Recently, OST1 was shown to phosphorylate *ICE1* to increase its stability. The knockout lines of OST1 were found to be defective in freezing tolerance whereas overexpression lines exhibited enhanced tolerance (Ding et al., 2015). Though OST1 protein was not previously observed to be cold-inducible our results suggests that *SnRK2.6* is up-regulated on exposure to cold stress in *B. juncea* siliques. It will be interesting to study whether SnRK2.6 phosphorylates ICE1 and possibly ICE2 under cold stress in *B. juncea* siliques. Traditionally the genes involved in cold acclimation process have been used to impart both chilling as well as freezing tolerance in plants. With the help of RNA-seq we identified genes that are up-regulated by chilling stress and potentially these genes can be utilized to introduce frost tolerance during pod filling stages in *B. juncea*. In addition to identifying homologs of CBF1-mediated cold stress signaling pathway genes, we also identified genes whose products have not yet been implicated in providing protection against low temperature stress. Further functional studies involving these genes will not only help us in extending our understanding on cold stress signaling but also pave way for designing frost hardy plants.

## Author contributions

MA conceived, designed and supervised the research work. AJ and SK-A participated in regular discussions for designing the wet lab experiments and analysis of the sequencing data. SS and VKR contributed equally to the study. SS performed self-pollination, stress treatment, RNA isolation, prepared RNA-seq libraries, and qPCR based expression analysis. VKR performed high throughput sequencing and analyzed the transcriptome data. SS, VKR, and MA wrote the manuscript. BJ performed the anatomical staging. AJ and SK-A provided critical inputs for data presentation. AJ, SK-A, SG, and AK critically reviewed the manuscript. All authors read and approved the final manuscript.

### Conflict of interest statement

The authors declare that the research was conducted in the absence of any commercial or financial relationships that could be construed as a potential conflict of interest.
