# Supplementary material for: De novo transcriptome profiling of cold-stressed siliques during pod filling stages in Indian mustard (Brassica juncea L.)
Source: Front Plant Sci. 2015 Oct 30;6:932. doi: 10.3389/fpls.2015.00932 (PMC4626631; doi:10.3389/fpls.2015.00932)
Supplement: Supplementary file 1 [file DataSheet1.DOCX]

To study the anatomical details of *B. juncea* var. Varuna over various stages of seed development, 20 siliques were harvested at a regular interval of 5 days between 5 to 30 days after pollination. Randomly selected 10 seeds each from 5, 10, 15, 20, 25 and 30 days after pollination were fixed in Karnovsky’s fixative for 24 h at 4^◦^C (Karnovsky, 1965). Fixed material was washed with sodium cacodylate buffer (0.2 M) and stored in the same. For anatomical studies, resin sectioning was performed (Feder and O'brien, 1968), Resin sections (3 to 5 µm thick) were prepared using a rotary microtome (AO Spencer, Model 820, USA) with glass knives. The glass knives were prepared by sing glass knife maker (LKB, USA). The resin embedded sections were stained with toluidine blue ‘O’ (TBO; Sigma) to study the structural details. Toluidine blue ‘O’ was prepared as per protocol described by O'Brien and McCully, 1981.

**Imaging**

The TBO stained sections were viewed under bright field microscope (Primo Star, Carl Zeiss, Germany) equipped with CANON Power shot G-10 camera and all photomicrographs were captured using Axio Vision 4 software. All images were processed and improved for photo plates with the help of Adobe Photoshop CS5 software (Adobe Systems Incorporated. 2010, California) without any manipulations.

**Supplementary Figure:** Longitudinal sections of fertilized ovule showing developmental stages after (A) 5 DAP, (B) 10 DAP, (C) 15 DAP**, (**D) 20 DAP, (E) 25 DAP and (F) 30 DAP in *B. juncea* var. Varuna.


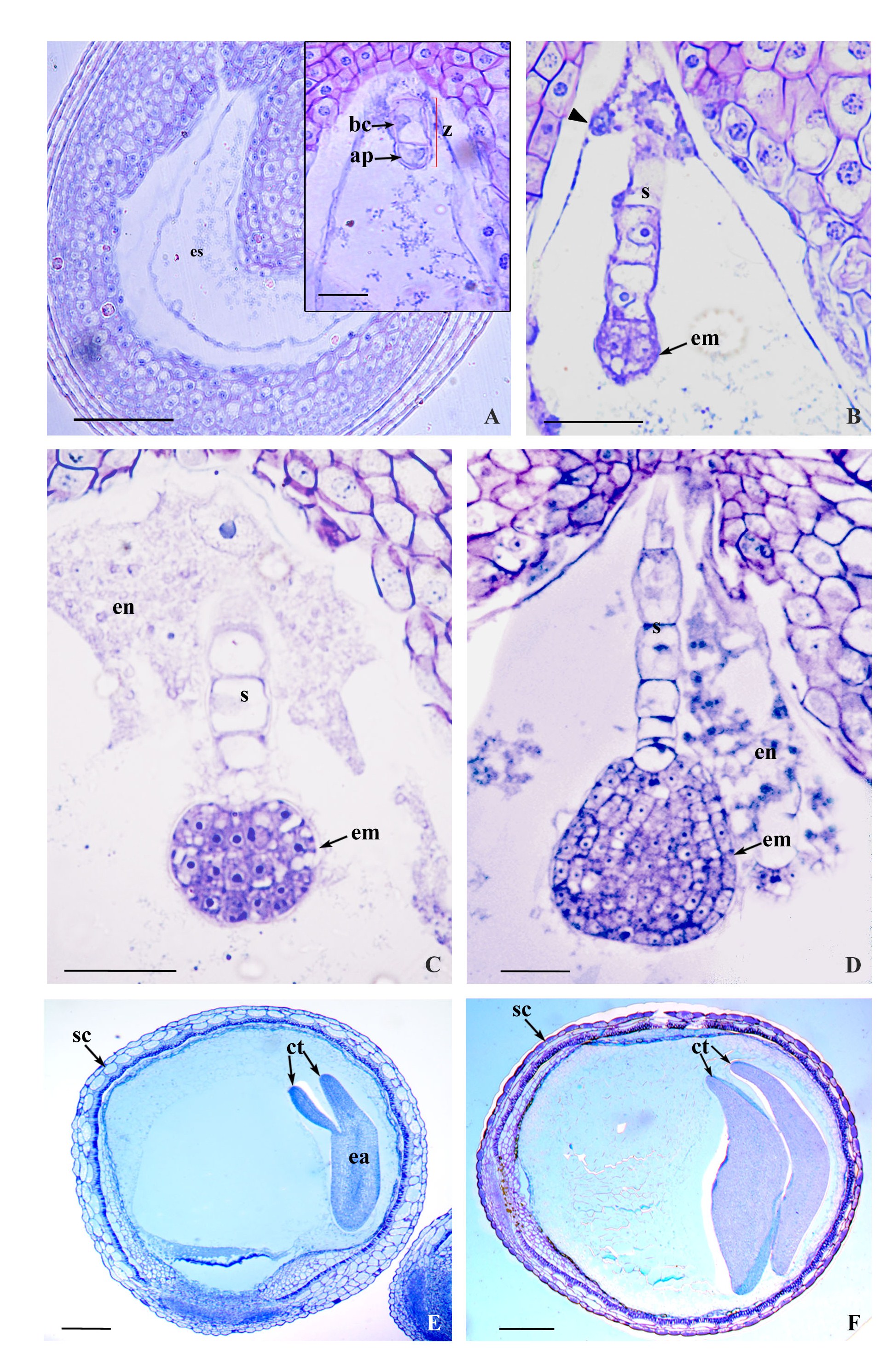


1. LS of ovule showed first division of zygote forming the apical cell and basal cell. Apical cell develops into the embryo, while basal cell forms suspensor to provide nutrition to the developing embryo**.**
2. LS of ovule showed first longitudinal division of embryo. Endosperm nuclei were also observed (marked by black arrow).
3. Globular stage of embryo and some remnants of degrading endosperm.
4. Heart shape embryo.
5. Torpedo stage of embryo.
6. Cotyledonary immature embryo.

**Abbreviations:** es, embryo sac; z, zygote; bc, basal cell; ap, apical cell; s, suspensor; em, embryo; en, endosperm; sc, seed coat; ct, cotyledons; ea, embryonal axis.

**Scale bar:** A=200 µm (inset =50 µm), B-D=50 µm, E=200 µm, F=500 µm
