# Supplementary material for: De novo transcriptome profiling of cold-stressed siliques during pod filling stages in Indian mustard (Brassica juncea L.)
Source: Front Plant Sci. 2015 Oct 30;6:932. doi: 10.3389/fpls.2015.00932 (PMC4626631; doi:10.3389/fpls.2015.00932)
Supplement: Supplementary file 7 [file Presentation1.PDF]

Supplementary Material

De novo transcriptome profiling of cold-stressed siliques during pod filling stages of Indian mustard (*Brassica juncea* L.)

Somya Sinha[1#], Vivek Kumar Raxwal[1,2#], Arun Jagannath[1], Surekha Katiyar-Agarwal[3], Shailendra Goel[1], Amar Kumar[1] and Manu Agarwal*[1]

[#]Authors contributed equally to this work.

***Correspondence:** Manu Agarwal, Department of Botany, North Campus, University of Delhi, Delhi-110007, India. agarwalm71@gmail.com; magarwal@botany.du.ac.in

1 Supplementary Datasheet

Supplementary Datasheet 1: Procedure for anatomical staging of embryo development in *B. juncea* var. Varuna and the figure of the various stages obtained.

Supplementary Datasheet 2: List of identified transcripts with their respective IDs, length, relative fold change, best BLASTx hit to protein database and gene ontologies.

Supplementary Datasheet 3: List of differentially expressed transcripts with their respective IDs and TMM-normalized FPKM expression values.

Supplementary Datasheet 4: Description of transcripts inducible at early stages of silique development in cold stress

Supplementary Datasheet 5: Description of transcripts inducible at all stages of silique development in cold stress

Supplementary Datasheet 6: Description of transcripts inducible at late stages of silique development in cold stress

2 Supplementary Figures and Tables

1.1 Supplementary Figures

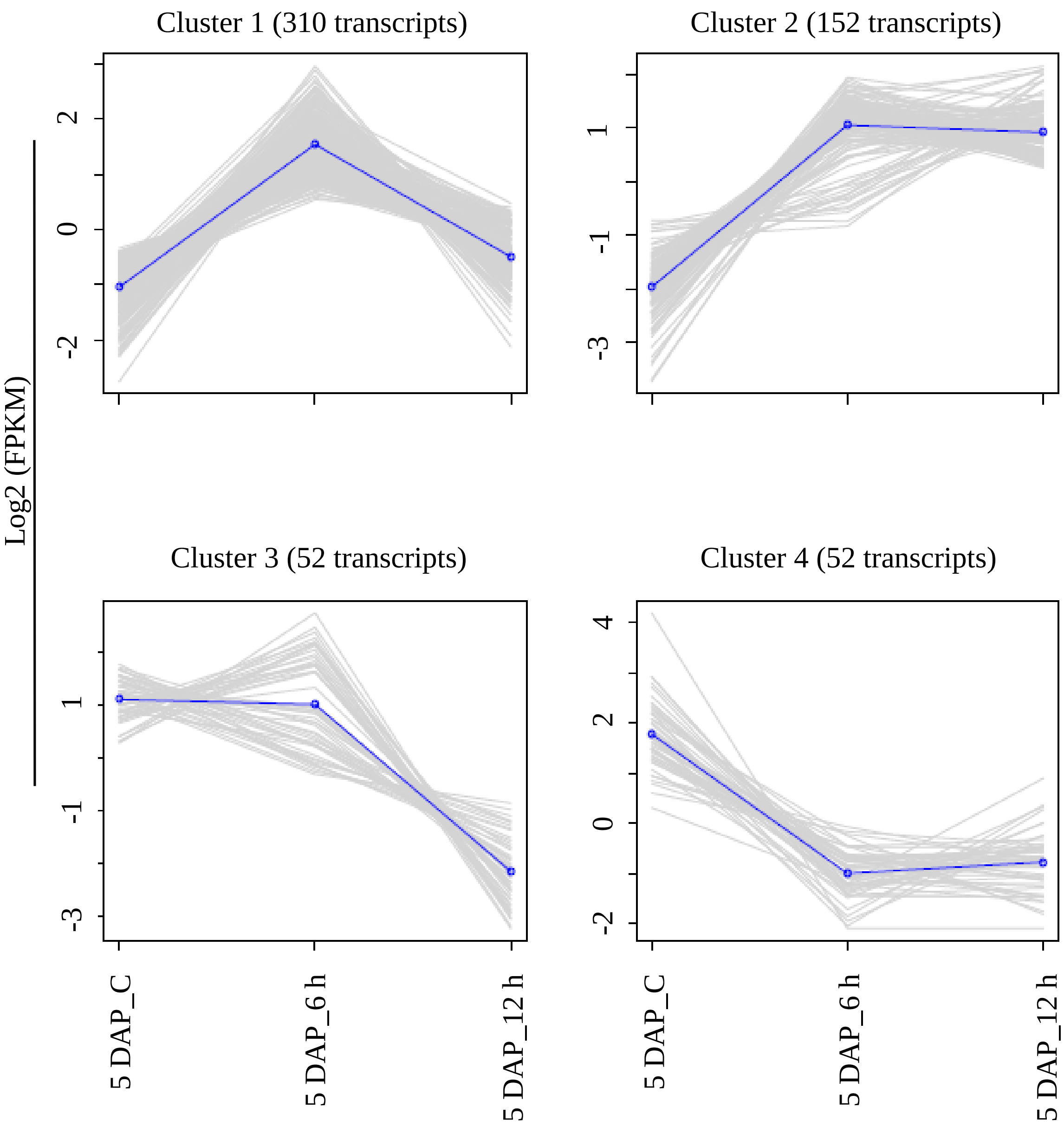

Supplementary Figure 1: Expression profile of transcripts in 5 DAP old *B. juncea* siliques exposed to cold stress. The transcripts showing similar expression profile in samples of 5 DAP (control and cold stress) were grouped into clusters. The average expression of each cluster was plotted in dark blue line whereas grey lines represent expression of individual transcripts.

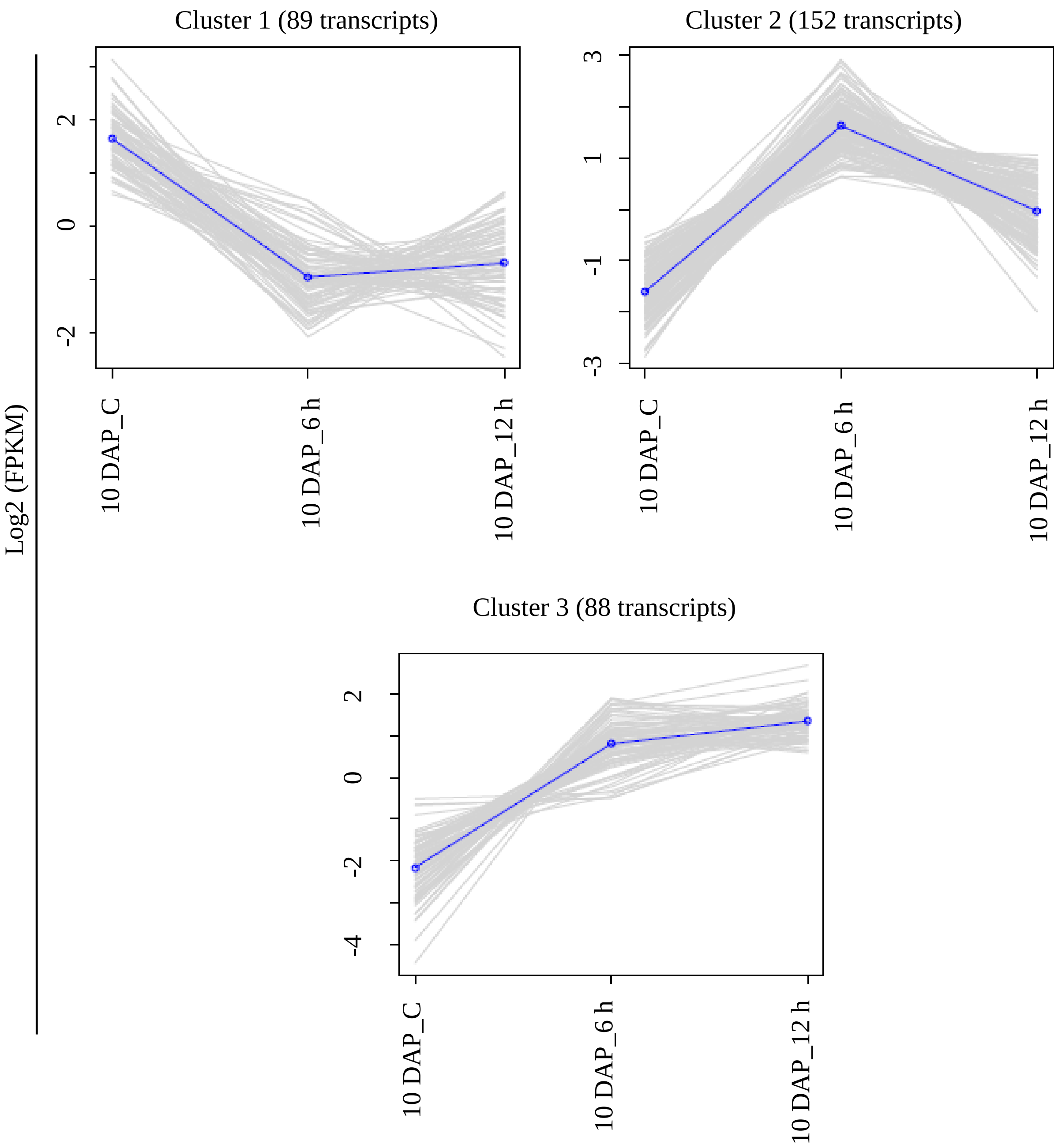

Supplementary Figure 2: Expression profile of transcripts in 10 DAP old *B. juncea* siliques exposed to cold stress. The transcripts showing similar expression profile in samples of 10 DAP (control and cold stress) were grouped into clusters. The average expression of each cluster was plotted in dark blue line whereas grey lines represent expression of individual transcripts.

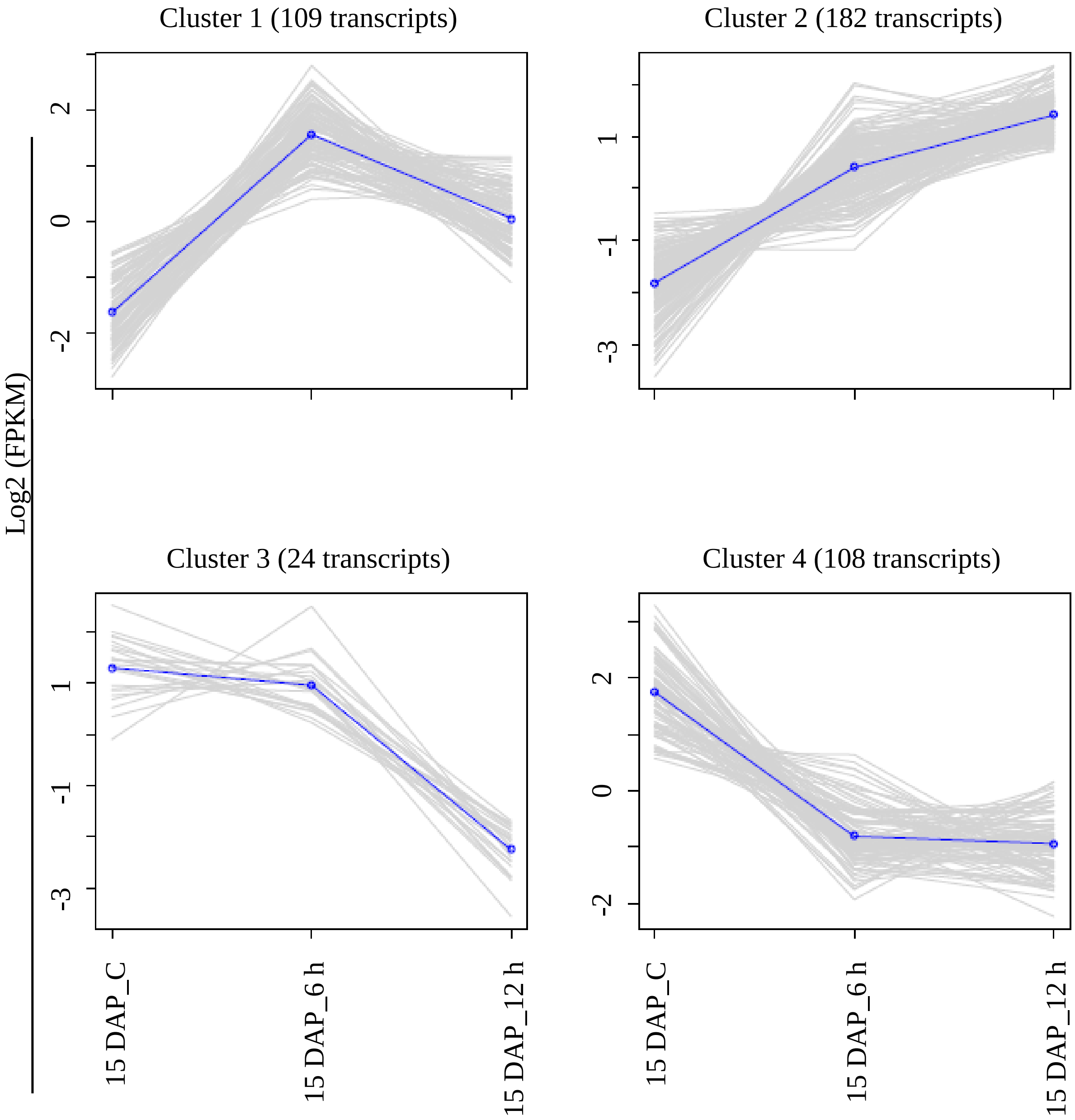

Supplementary Figure 3: Expression profile of transcripts in 15 DAP old *B. juncea* siliques exposed to cold stress. The transcripts showing similar expression profile in samples of 15 DAP (control and cold stress) were grouped into clusters. The average expression of each cluster was plotted in dark blue line whereas grey lines represent expression of individual transcripts.

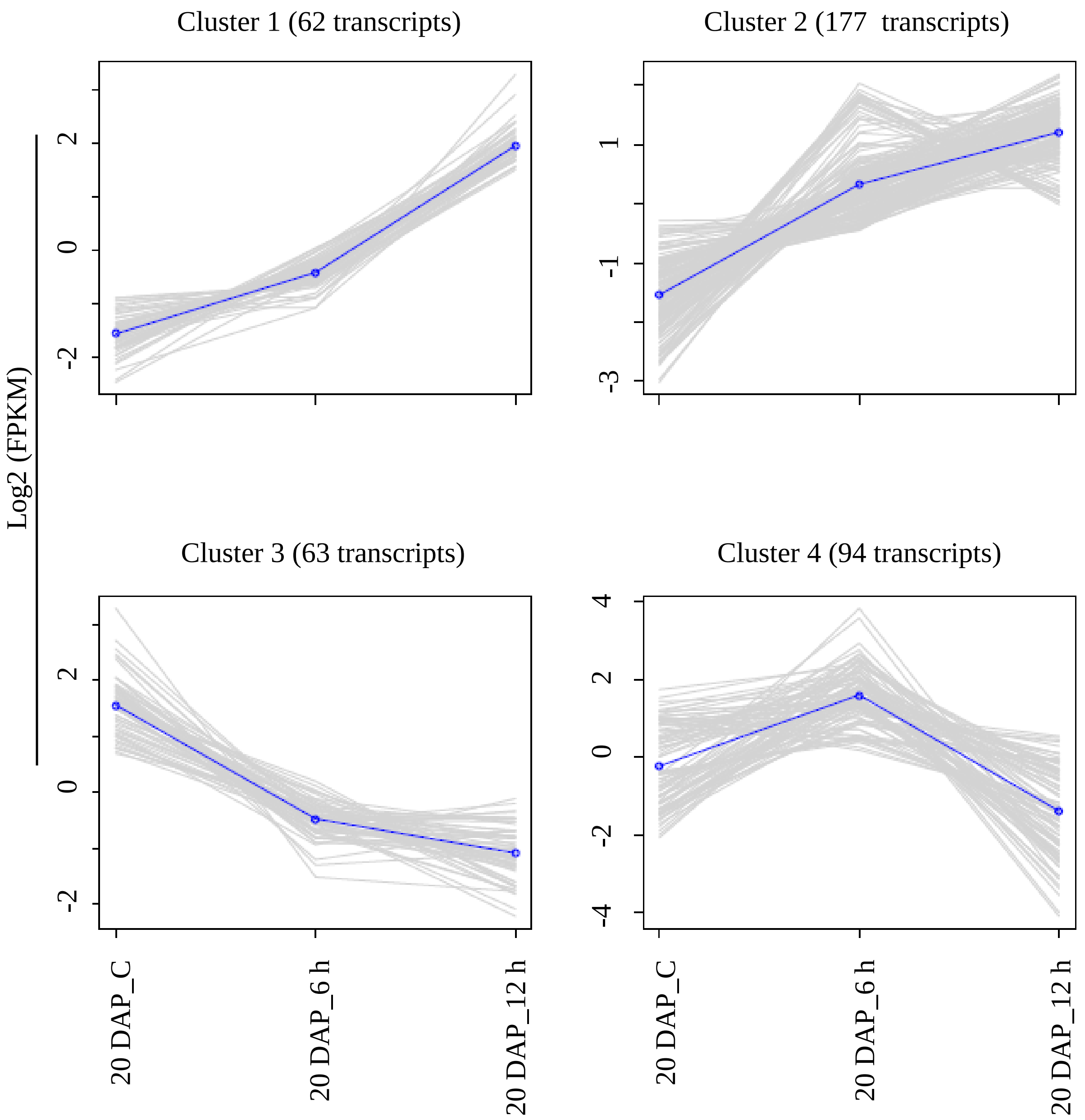

Supplementary Figure 4: Expression profile of transcripts in 20 DAP old *B. juncea* siliques exposed to cold stress. The transcripts showing similar expression profile in samples of 20 DAP (control and cold stress) were grouped into clusters. The average expression of each cluster was plotted in dark blue line whereas grey lines represent expression of individual transcripts.

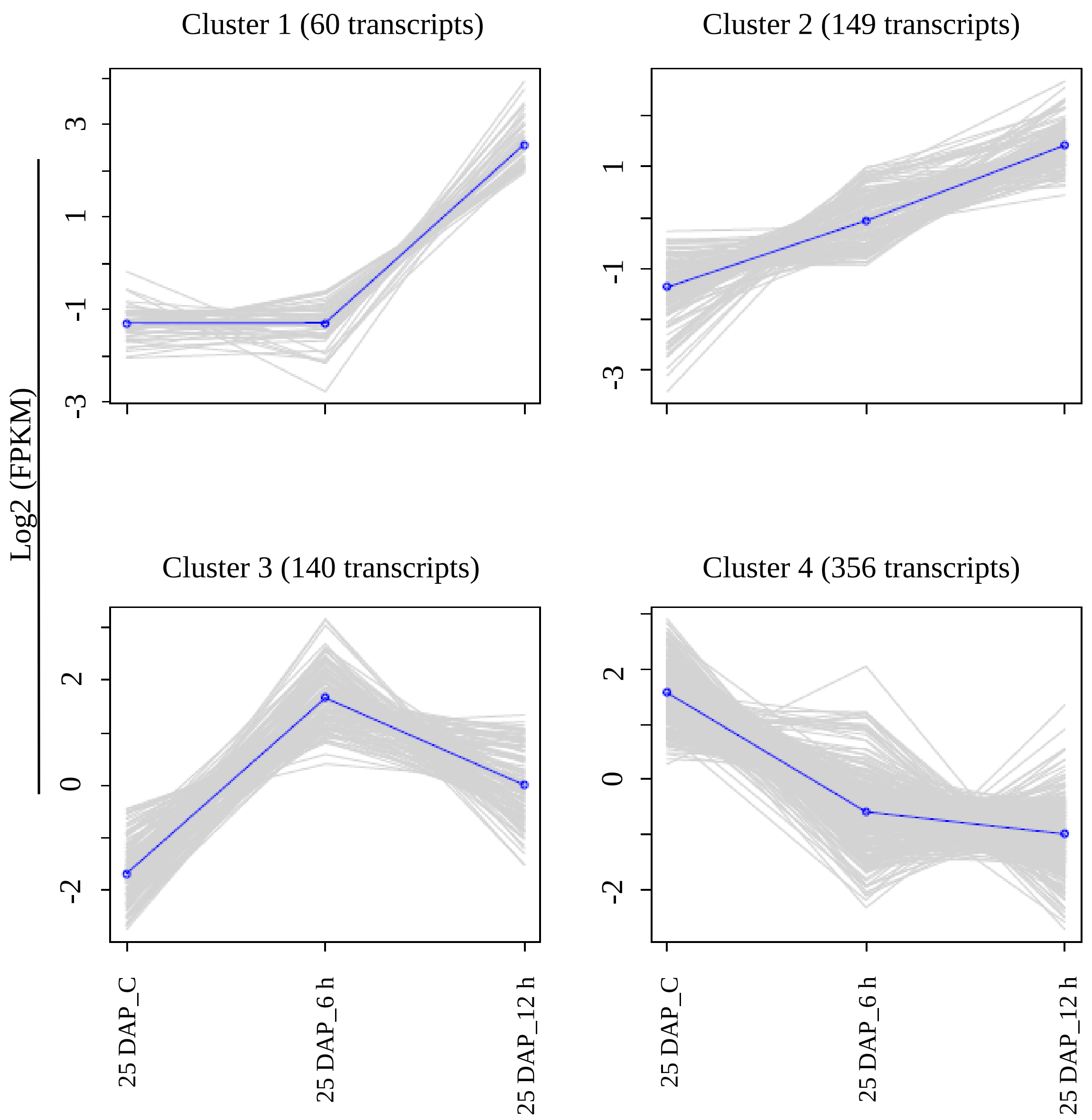

Supplementary Figure 5: Expression profile of transcripts in 25 DAP old *B. juncea* siliques exposed to cold stress. The transcripts showing similar expression profile in samples of 25 DAP (control and cold stress) were grouped into clusters. The average expression of each cluster was plotted in dark blue line whereas grey lines represent expression of individual transcripts.

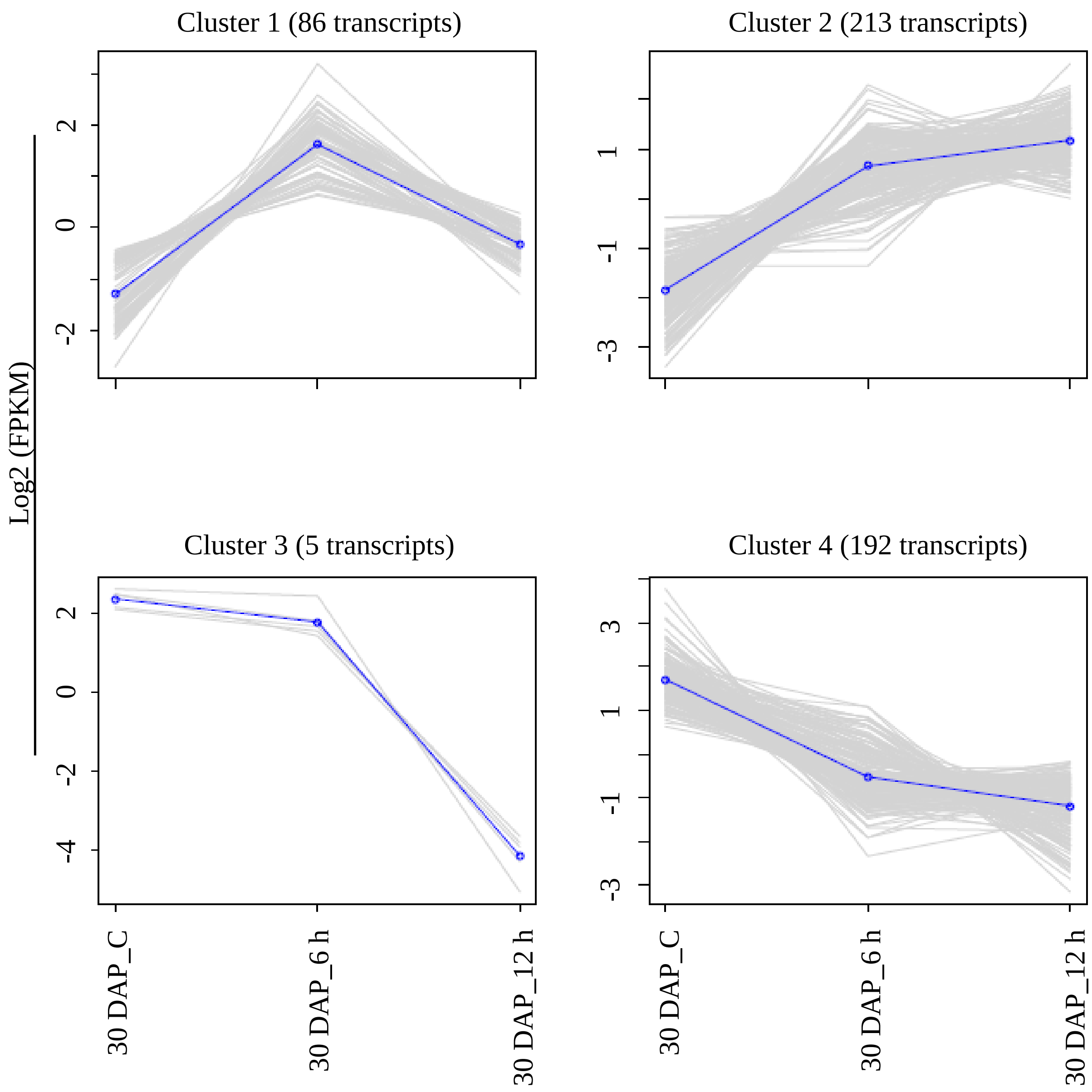

Supplementary Figure 6: Expression profile of transcripts in 30 DAP old *B. juncea* siliques exposed to cold stress. The transcripts showing similar expression profile in samples of 30 DAP (control and cold stress) were grouped into clusters. The average expression of each cluster was plotted in dark blue line whereas grey lines represent expression of individual transcripts.

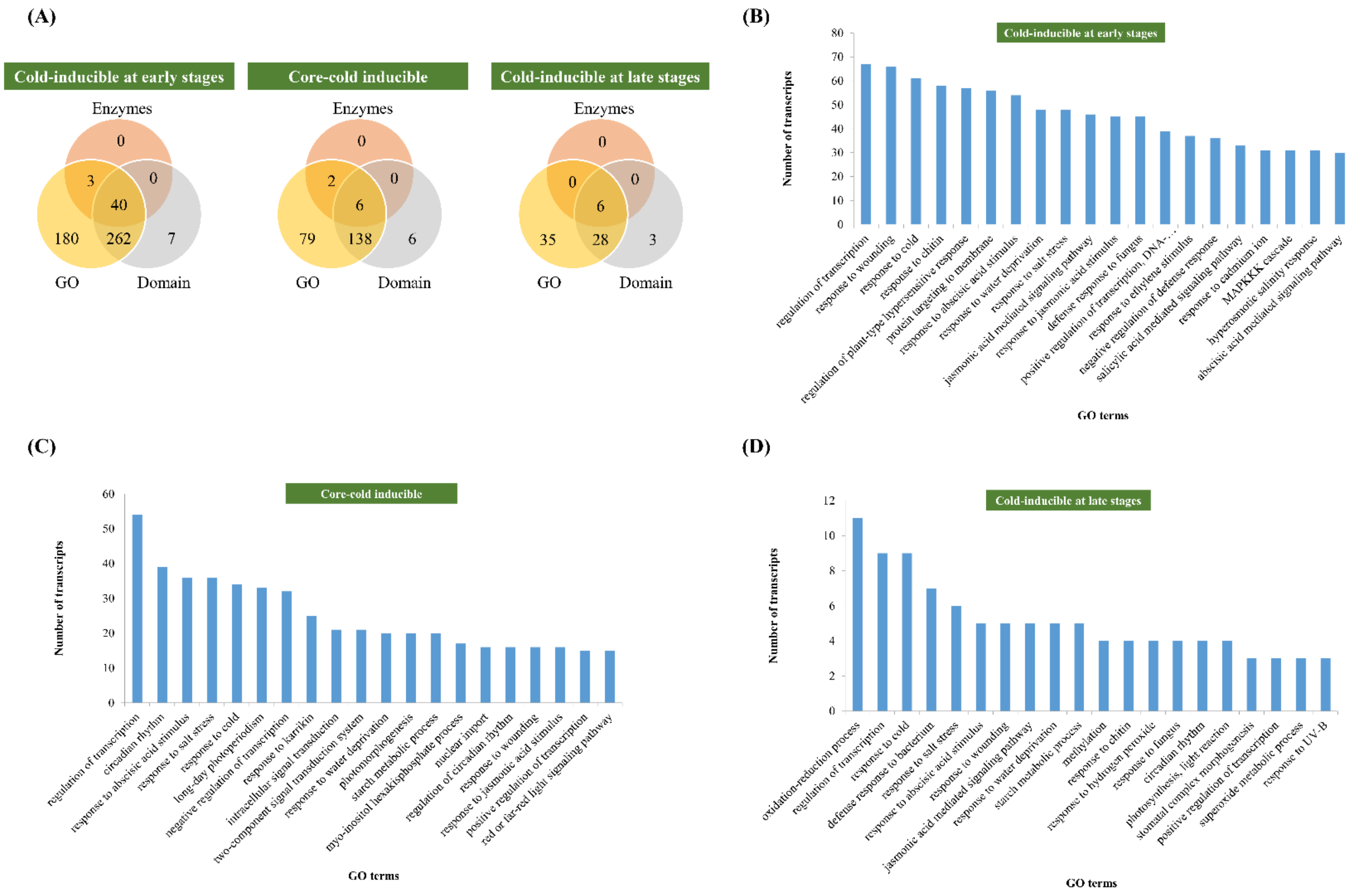

Supplementary Figure 7: (A) Distribution of annotated cold-inducible *B. juncea* transcripts based on their databases hits. The LAST search was used to identify transcripts in NCBI nr database whose output was further annotated with Blast2GO for assigning gene ontological terms to transcripts. To identify transcripts similar to enzyme category PRIAM database was utilized whereas RPS blast was used to identify conserved domain by mapping to Pfamv26 database. The transcripts were classified on the basis of gene ontologies and 20 most enriched GO terms from the biological process category in three subsets of cold-inducible transcripts: early (**B**), core **(C)** and late stages **(D)** of silique development are presented.

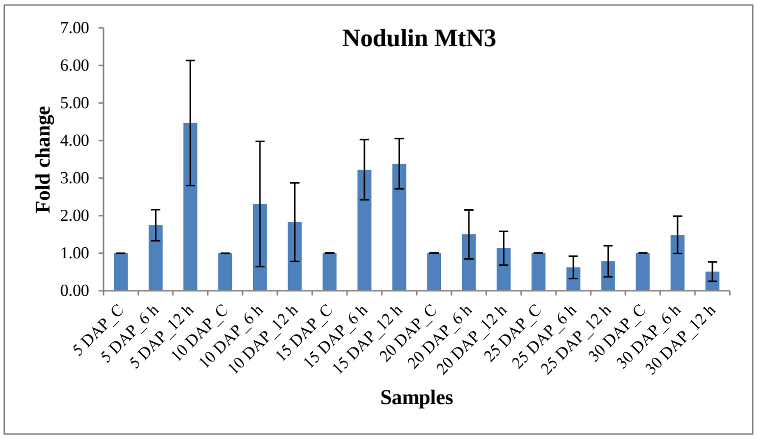

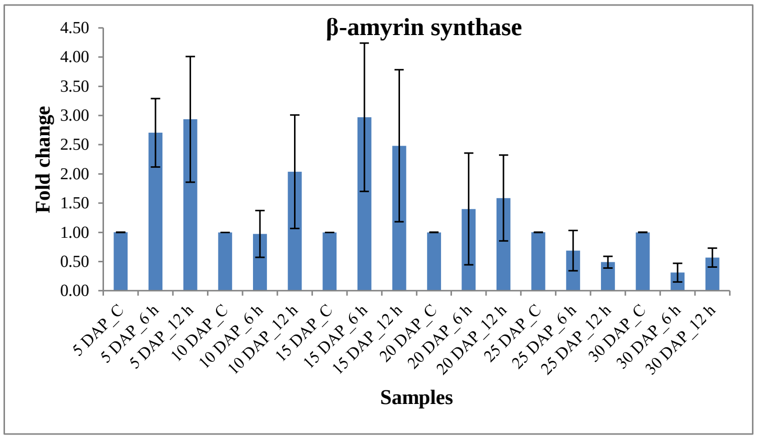

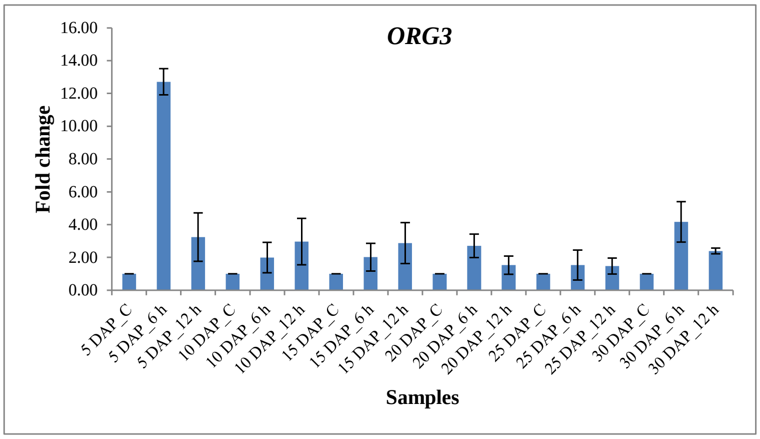

Supplementary Figure 8: Relative abundance of selected transcripts specific to early cold-inducible subsets as determined by qPCR. Quantitative PCR was performed with total RNA isolated from *B. juncea* siliques (5 DAP-30 DAP) subjected to cold stress (6 h and 12 h) and corresponding control samples. The expression analysis was done using three biological and two technical replicates. The error bar denotes standard error among three independent biological replicates.

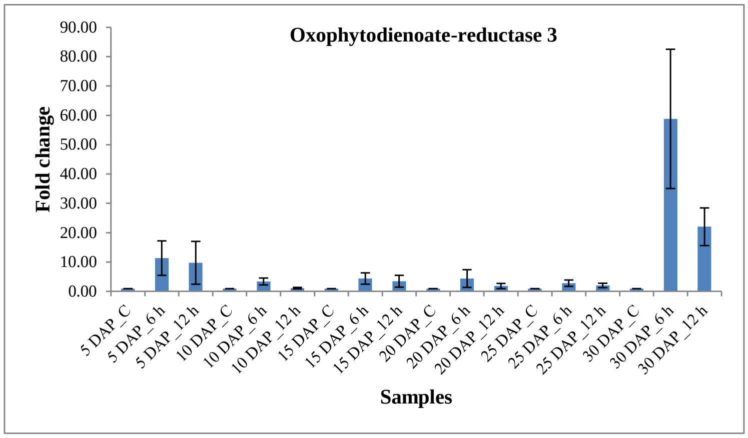

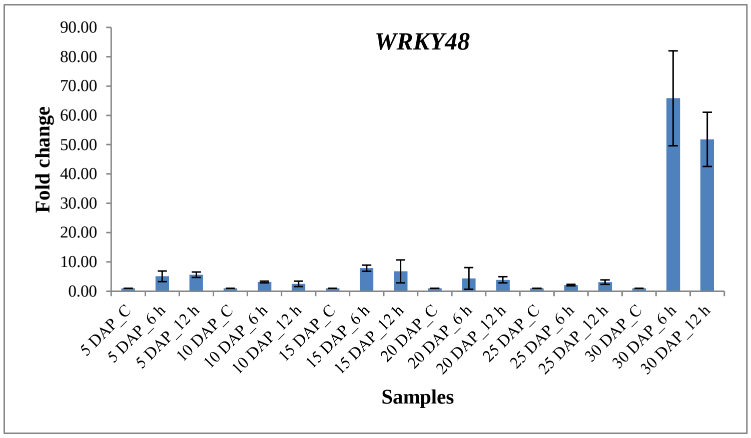

Supplementary Figure 9: Relative abundance of selected transcripts specific to late cold-inducible subsets as determined by qPCR. Quantitative PCR was performed with total RNA isolated from *B. juncea* siliques (5 DAP-30 DAP) subjected to cold stress (6 h and 12 h) and control samples. The expression analysis was done using three biological and two technical replicates. The error bar denotes standard error among three independent biological replicates.

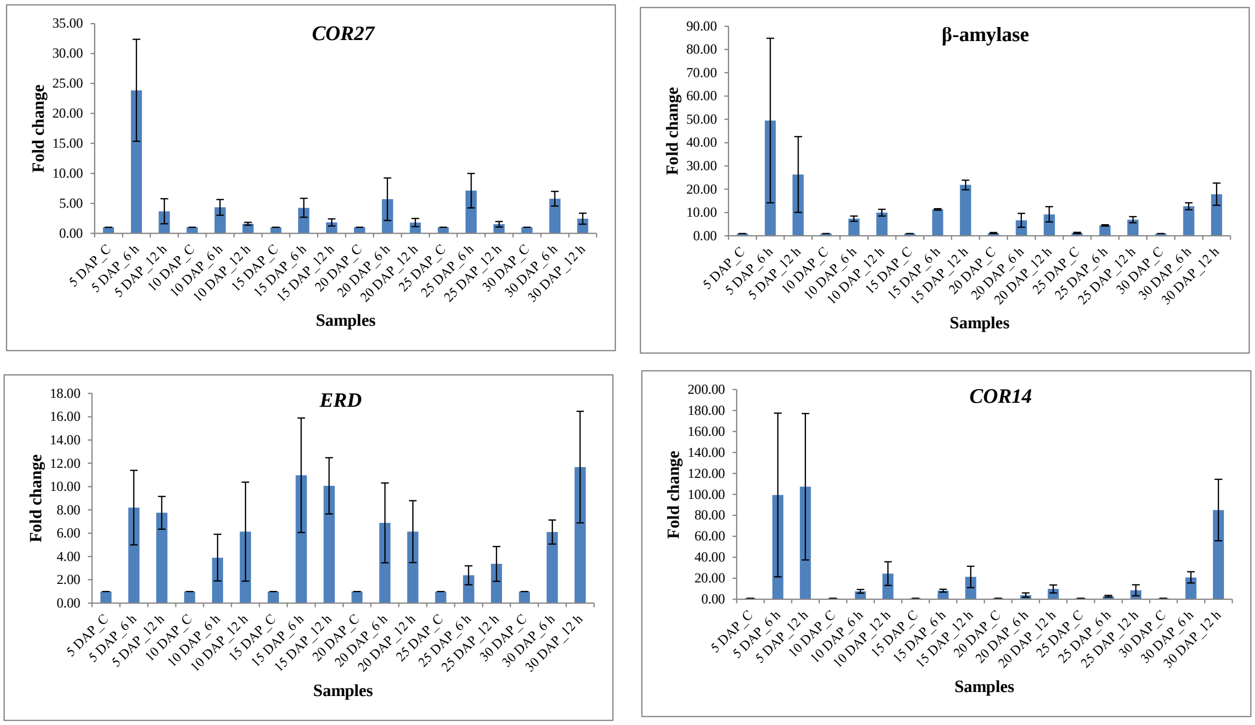

Supplementary Figure 10: Relative abundance of selected transcripts specific to core-cold inducible subsets as determined by qPCR. Quantitative PCR was performed with total RNA isolated from *B. juncea* siliques (5 DAP-30 DAP) subjected to cold stress (6 h and 12 h) and control samples. The expression analysis was done using three biological and two technical replicates. The error bar denotes standard error among three independent biological replicates.

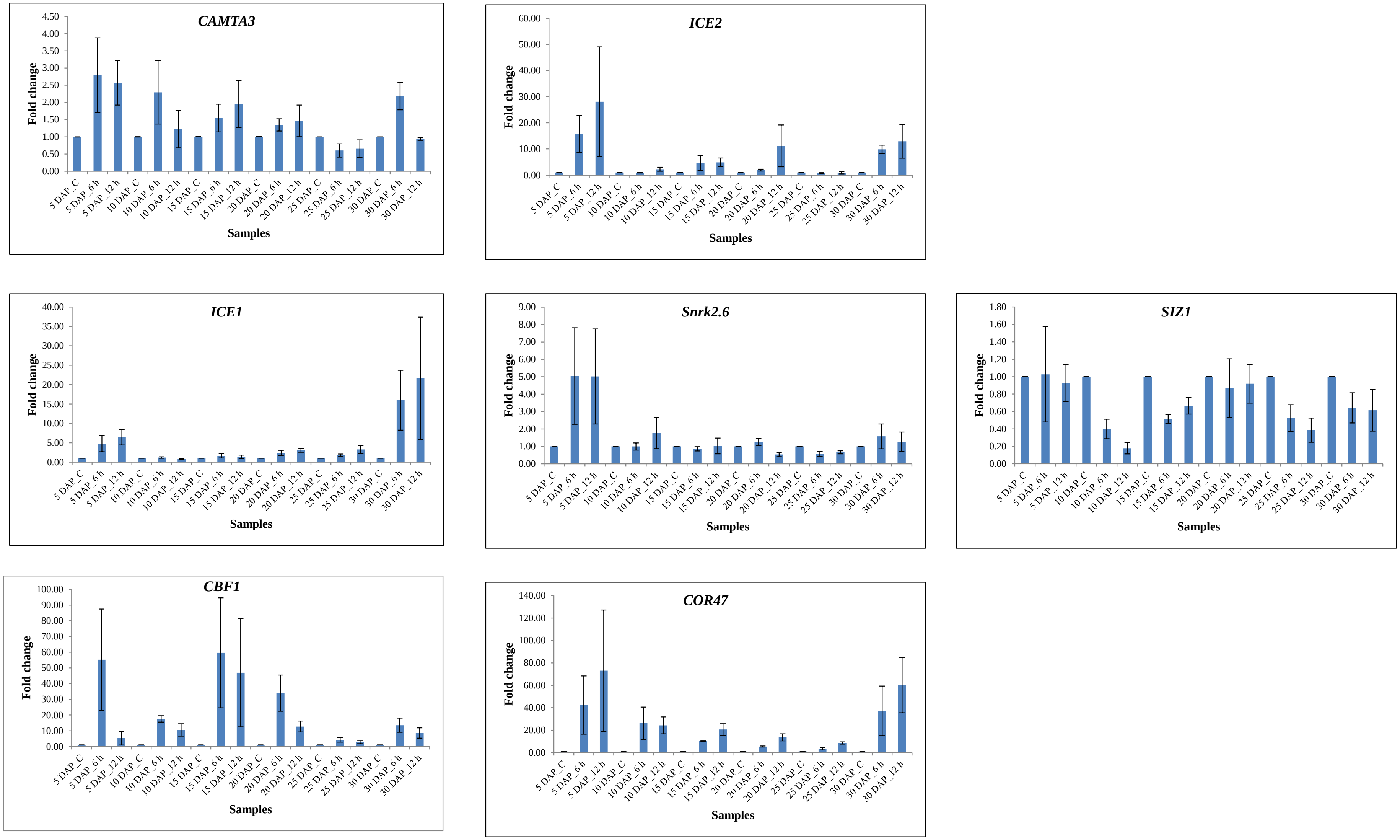

Supplementary Figure 11: Relative abundance of selected transcripts specific to cold stress pathway as determined by qPCR. Quantitative PCR was performed with total RNA isolated from *B. juncea* siliques (5 DAP-30 DAP) subjected to cold stress (6 h and 12

h) and control samples. The expression analysis was done using three biological and two technical replicates. The error bar denotes standard error among three independent biological replicates.

3 Supplementary Tables

Supplementary Table 1: List of primers employed for qPCR analysis.

S. No.	Transcript ID	Hits to NCBI nr-database	Forward primer (5'-3')	Reverse primer (5'-3')
1	c64940_g1_i1	β-amyrin synthase	CCTTTACGCATAGCAAGACT	ATATACGCCACATGGTTCG
2	c64943_g1_i1	Nodulin MtN3 family protein	ACTCCACACTTCTTGTTCGTATAA	ACGTGAGAAAGTTCTCGG
3	c57701_g1_i2	ORG3-like transcription factor	GATCAGAGGCTGGAAGAC	GTCACAATGCTAATGAGCG
4	c66940_g1_i1	Oxophytodienoate-reductase 3	CCTCGGTACCACGCTTATGGGC	GCTGAATGGCTTGCATACCAAGTT
5	c69010_g2_i2	*WRKY48* transcription factor	AACGGTAATAGCTTCTGGG	TCTTGACGACGGTTATAGGTG
6	c64594_g1_i1	*COR 27*	CTGGCAGAAGATGAATGTG	ATGGACTCCTAAGAAACTCG
7	c69711_g17_i1	*COR 14*	AGAAGTCGTTGATCTACGC	TCTTGTCTGTCACGTAATCTGAA
8	c70856_g2_i2	Putative β-amylase	GCTGGTGGAGTTTGTGAAGAA	ATAAAGGTCACTCCCTGTTG
9	c66527_g1_i2	Early responsive to dehydration	AGAGGATCTTGGAGGATTACA	CATCCAACACCTCTTCCC
10	c106397_g1_i1	*CBF1*	GAGATTTCAACAATGACCTCATTT	TTCGGACAATACTCCGCCTAA
11	c71663_g1_i1	*ICE1*	GCGATGATGGAGATATGGAT	AACACTCTCAGAAGCTTTACC
12	c154106_g1_i1	*ICE2*	TGCTCAGATCAGTTGTCCC	GAGCTCTTTAAGATAATCAATGGC
13	c71974_g1_i1	*CAMTA3*	TGGTCTTGCATGTTTGGG	TGCATAGGAGCAACACACT
14	c72891_g1_i6	*SIZ1*	GGAGGTGGAGATGATAATGC	CCGCTCATAGGACAACGA
15	c69724_g2_i1	*SnRK2.6*	AGATGTTTGGTCTTGTGGG	TGAAATTCTTAGGCTCATCAGG
16	c56112_g1_i1	*COR47*	CTTCACCAGAAGCACGAG	GAAGAGCTGTTGGATCGG
